# Inhibiting Glutaminase Exerts Opposite Effects on Ovariectomy-Induced and Age-Related Reductions in Murine Bone Mass

**DOI:** 10.14336/AD.2024.0201

**Published:** 2024-02-01

**Authors:** Qian Guo, Hongjian Zhao, Zijian Dong, Haozhe Cheng, Meipeng Zhu, Zhong Fang

**Affiliations:** Department of Orthopedic Surgery and Biological Engineering and Regenerative Medicine Center, Tongji Hospital, Tongji Medical College, Huazhong University of Science and Technology, Wuhan, China

**Keywords:** glutaminase, osteoclast differentiation, osteogenic differentiation, age-related osteoporosis, ovariectomy

## Abstract

Recent studies have provided links between glutamine metabolism and bone remodeling, but little is known about its role in primary osteoporosis progression. We aimed to determine the effects of inhibiting glutaminase (GLS) on two types of primary osteoporosis and elucidate the related metabolism. To address this issue, age-related and ovariectomy (OVX)-induced bone loss mouse models were used to study the in vivo effects of CB-839, a potent and selective GLS inhibitor, on bone mass and bone turnover. We also studied the metabolic profile changes related to aging and GLS inhibition in primary bone marrow stromal cells (BMSC) and that related with OVX and GLS inhibition in primary bone marrow-derived monocytes (BMM). Besides, we studied the possible metabolic processes mediating GLS blockade effects during aging-impaired osteogenic differentiation and RANKL-induced osteoclast differentiation respectively via in vitro rescue experiments. We found that inhibiting GLS via CB-839 prevented OVX-induced bone loss while aggravated age-related bone loss. Further investigations showed that effects of CB-839 treatment on bone mass were associated with alterations of bone turnover. Moreover, CB-839 treatment altered metabolic profile in different orientations between BMSC of aged mice and BMM of ovariectomized mice. In addition, rescue experiments revealed that different metabolic processes mediated glutaminase blockade effects between aging-impaired osteogenic differentiation and RANKL-induced osteoclast differentiation. Taken together, our data demonstrated the different outcomes caused by CB-839 treatment between two types of osteoporosis in mice, which were tightly connected to the suppressive effects on both aging-impaired osteoblastogenesis and OVX-enhanced osteoclastogenesis mediated by different metabolic processes downstream of glutaminolysis.

## INTRODUCTION

The adult bone is undergoing continuous remodeling. During bone remodeling, bone cell progenitors proliferate and differentiate into osteoblasts or osteoclasts, which deposit extracellular matrix followed by mineralization or dissolve the organic and inorganic matrix. The above processes are highly demanding for biosynthesis and bioenergy [1]. While the cell signals and transcriptional factors that control the differentiation and function of bone-forming osteoblasts and bone-resorbing osteoclasts have been extensively studied, the metabolic features are only beginning to be characterized during the past two decades [1]. To date, the majority of studies on bone tissue metabolism focused on glucose metabolism, which is the main nutrient consumed by both osteoclasts and osteoblasts [2, 3]. However, bone tissue also uptakes a large amount of other postprandial nutrients such as lipids and amino acids, which were recently identified to be transcriptionally regulated and tightly correlated with bone turnover [4, 5]. Unlike glucose, the functional roles of downstream metabolic pathways for these nutrients in bone tissue are not well understood.

Glutamine is the most abundant amino acid derivative in circulation with multiple metabolic roles [6]. Besides contribution to mitochondrial energy production via α-ketoglutarate (α-KG), glutamate can be utilized for amino acid and nucleotide biosyntheses and is also considered as an important precursor for glutathione (GSH) biosynthesis [7]. The intracellular glutamine catabolism (glutaminolysis) starts when glutaminase (GLS) deaminates glutamine to glutamate, which is the most critical step to fulfill the diverse functions of glutamine. Increasing studies have highlighted the demand for glutamine in bone since Biltz et al. firstly reported an active metabolic status of glutamine in isolated long bones and calvaria [8]. Recently, through genetic and metabolic approaches, studies have suggested an important regulatory role of GLS-dependent glutaminolysis in bone remodeling: in the aspect of bone formation, GLS promotes osteogenic lineage allocation and supports proliferation and osteoanabolic function of osteoprogenitors [9, 10]; in the aspect of bone resorption, glutaminolysis is also required for osteoclast differentiation [11, 12], while the intermediate metabolite α-KG which can promote proliferation and osteogenic potential of bone marrow mesenchymal stem cells [13], is proved to negatively interfere with osteoclast differentiation [14]. Additionally, previous researches have also reported the effects of glutamine-related anabolic therapies on various bone diseases, including accelerating fracture healing, diminishing fundectomy-induced osteopenia and glucocorticoid-induced bone loss via animal models [15-17]. Based on these evidences, it is clear that metabolic status of glutamine can not only be used as a biomarker of bone health but also as a potential therapeutic target.

The decreased bone formation upon aging and the excessive bone resorption caused by estrogen deficiency, often seen in the elderly and postmenopausal women respectively, commonly break the balance and are associated with bone loss or reduced bone quality [18, 19]. Interestingly, two independent researches based on different populations have both observed significant associations between serum glutamine level and bone mineral density, although it is unclear if the altered glutamine metabolism is due to osteoporosis or the driver of osteoporosis [20, 21]. Due to this, it is possible that the altered glutamine metabolism plays a role during the development of primary osteoporosis. However, given that the essential roles of glutaminolysis in bone cells have been gradually revealed [9-14], there is no animal study to discuss the therapeutic outcome of targeting GLS during progressive bone loss resulted from aging or estrogen deficiency.

In mammals, the GLS enzymes include two isoforms, GLS1 or kidney-type GLS widely and highly expressed in most nonhepatic tissues, and GLS2 or liver-type GLS primarily restricted to adult liver, encoded by two paralogous genes [22, 23]. CB-839 is a specific, potent and orally available first-in-class inhibitor of GLS1 [24]. And most importantly, CB-839 is the only GLS inhibitor currently being evaluated in clinical trials for efficacy and safety in a variety of pathological processes known to be driven by glutamine metabolism except osteoporosis [25, 26]. Here, we explored the effects of CB-839 treatment on the progression of primary osteoporosis via both ovariectomy (OVX)-induced and age-related bone loss mouse models. Interestingly, we have found that with the same dose, route, frequency and duration of administration, both inhibiting GLS by CB-839 and knocking down GLS1 via adenovirus significantly ameliorated OVX-induced bone loss but deteriorated age-related osteoporosis in mice.

## MATERIALS AND METHODS

### Chemical compound and reagents for treatments

CB-839 (S76655) was obtained from Selleck Chemicals. Glutathione ester ethyl (GSH-EE, G1404), 100× MEM Non-essential amino acids (NEAA, M7145) and 100× EmbryoMax nucleotide mixture (nucleo, ES-008) were bought from Sigma-Aldrich. We determined dose, route and frequency of CB-839 administration in animal experiments following those applied in a previous study which were proved to effectively decrease GLS activity in mice [26]. For in vitro treatments, CB-839 (8 mM) and GSH-EE (1M) were both dissolved in DMSO and stored as stock solutions and were diluted by medium for the experiments. Medium supplemented with 1× NEAAs, 1× nucleotide mixture or 1mM GSH-EE solution was applied to treat cells in certain rescue experiments. Recombinant soluble murine receptor activator of NF-κB ligand (RANKL) and macrophage colony-stimulating factor (M-CSF) were bought from R&D Systems.

### Construction of adenovirus and in vitro transduction

For glutaminase 1 (GLS1) knockdown, adenovirus expressing short hairpin RNAs (shGLS1) against transcripts of murine *Gls1* (NM_001081081.2 and NM_001113383.1) was purchased from Vigene Biosciences. Four shRNAs designed towards different regions of above two transcripts were packaged in one vector and the sequences were: shRNA1, 5’- GGGAAC TGAGTATGTACATCGTTCAAGAGACGATGTACATACTCAGTTCCCTTTTTT-3’; shRNA2, 5’-GGAGCA ATTGTTGTGACTTCTTTCAAGAGAAGAAGTCAAACAATTGCTCCTTTTTT-3’; shRNA3, 5’-GCATTC TTGTGGCATGTATGATTCAAGAGATCATACATGCCACAAGAATGCTTTTTT-3’; shRNA4, 5’-GCAAC AGTGTTAAGGGAATTCTTCAAGAGAGAATTCCCTTAACACTGTTGCTTTTTT-3’. Adenovirus expressing scrambled shRNAs (SC) were used as control.

For mitochondrial pyruvate carrier (MPC) overexpression, coding sequences of wildtype murine *mpc1* (NM_018819.4) and *mpc2* (NM_027430.2) were cloned and packaged into one adenoviral (pAdM) vector and plasmid-based adenovirus packaging was then completed in Vigene Biosciences. Adenovirus expressing empty vector (EV) was used as control. Adenovirus particles were used to transduce primary BMM or BMSC with HiTransG (Shanghai GeneChem Company).

### Animal models and study design

8-week-old wildtype C57BL6/J female mice were supplied by Beijing Huafukang Bioscience Co. Ltd. (Beijing, China). 2-month-old and 14-month-old wildtype C57BL6/J mice of both sexes were obtained from Vital River Laboratory Animal Technology Co. Ltd. (Beijing, China). All animal experiments were performed in Experimental Animal Center of Tongji Hospital (Wuhan, China) strictly following the guidelines for care and use of laboratory animals, and all experimental protocols involving animals in this study were reviewed and approved by the Institutional Animal Ethics Committee of Tongji Hospital (No. TJH-202103001). Wild-type mice were housed in (5 mice per cage) a specific pathogen-free (SPF) animal laboratory with controlled conditions (18-22 °C and a 12:12 h light-dark cycle) and allowed free access to clean food and water. Mice of the same sex were caged together.

CB-839 was dissolved and formulated as a solution at 20 mg/mL in vehicle which consisted of 10 mmol/L citrate solution and 25% hydroxypropyl-β-cyclodextrin (PH = 2.0). For the study of OVX-induced bone loss, 8-week-old C57BL6/J female mice were housed in the SPF facility for 2 weeks before anesthetized by an intraperitoneal injection of pentobarbital for bilateral ovariectomy or sham operation. Later, mice were randomly divided into four groups (sample size n = 7 for each group) followed by specified treatment started 2 weeks after surgery (12 weeks of age): sham-operated mice treated with vehicle (SHAM group) or 200mg/kg CB-839 (SHAM + CB-839 group), and ovariectomized mice treated with vehicle (OVX group) or 200 mg/kg CB-839 (OVX + CB-839 group). Mice were weighted weekly and treated with CB-839 (200 mg/kg body weight) or same volume of vehicle via intragastric administration twice daily (every 12 h). After 8 weeks of treatment, mice were euthanized by cervical dislocation after being anesthetized and long bones including femurs and tibias were excised.

For the study of age-related bone loss, 2-month-old and 14-month-old C57BL6/J male (or female) mice were housed in the SPF facility for 2 months before treatments. Then young and aged male (or female) mice were randomly divided into four groups (sample size n = 6 for each group) followed by specified treatment in each group: young mice treated with vehicle or CB-839, and old mice treated with vehicle or CB-839. Treatment of either vehicle or CB-839 was performed in the same way (same dose, route, frequency and duration of administration) as that described in the above OVX-induced bone loss study. After 8 weeks of treatment, sample collections of 6-month-old and 18-month-old male (or female) mice were done likewise.

For the study of in vivo GLS1 knockdown, OVX-induced and age-related bone loss mouse models were constructed in the same manner firstly. Then, the adenovirus encoding shGLS1 or the control adenovirus encoding scrambled shRNAs were injected intravenously into the murine tail vein at the dose of 1 × 10^8^ plaque forming units and the injection was administered once per week. After 8 weeks of treatments, bone samples were collected.

### Isolation of bone marrow stromal cells (BMSC) and bone marrow-derived monocytes (BMM), and extraction of intracellular metabolites

For every mouse involved in above animal studies, we firstly isolated bone marrow cells as previously described [27]. Briefly, after being dissected and cut in order to expose marrow cavity, freshly isolated femur and tibia bones from C57BL6/J mice were flushed with PBS containing 5% fetal bovine serum (FBS) and bone marrow cells were suspended in the solution. Then red blood cells were depleted from the cell suspensions via red blood cell lysis buffer rapidly and the remaining cells were subsequently centrifuged at 300 G. The pelleted bone marrow cells (total BMC) were subsequently used to isolate BMSC and BMM.

BMSC isolation from total BMC and collection were performed as described by Maridas et al. in 2018 [28]. The non-adherent hematopoietic stem cells (HSC) in the former step were transferred to Ficoll-Hypaque (GE Healthcare) density gradient to collect BMM from the buffy coat layer. Both BMSC and BMM were snap frozen in liquid nitrogen suddenly after collection and sent to Novogene Co., Ltd (Beijing, China) in dry ice.

The extraction of intracellular metabolites was performed in Novogene Co., Ltd (Beijing, China). Briefly, cell samples from the same group were homogenized and underwent methanol extraction procedure, then the intracellular metabolite extracts were split into aliquots and injected into LC-MS/MS system analysis.

### Metabolite profiling via untargeted liquid chromatography-mass spectrometry/mass spectrometry (LC-MS/MS)

Untargeted LC-MS/MS analysis was performed for the intracellular metabolite extracts of bone marrow samples via ultrahigh performance liquid chromatography/ mass spectrometry (UHPLC/MS) in Novogene Co., Ltd (Beijing, China). The major instruments used in the analysis were a Vanquish UHPLC system (ThermoFisher, Germany) combined with an Orbitrap Q Exactive^TM^ HF mass spectrometry (ThermoFisher, Germany). A Hypesil Goldcolumn (100 × 2.1 mm, 1.9 μm) was applied to achieve the sample separation using a 17min linear gradient at a 0.2 mL/min flow rate. The mobile phases for positive polarity mode were 0.1% formic acid in water (eluent A) and methanol (eluent B), and that for negative polarity mode were 5 mM ammonium acetate (pH = 9.0, eluent A) and methanol (eluent B). The elution gradient was set as previously described [29]. Mass spectrometry was operated in both negative and positive polarity modes and the parameters and conditions were set following the previous study [29].

The raw data files generated by the LC-MS/MS system were analyzed by the Compound Discoverer 3.1 (CD3.1, ThermoFisher) in order to acquire peak alignment, peak picking, and quantitation for each metabolite. Parameters were set as follows: actual mass tolerance, 5 ppm; retention time tolerance, 0.2 minutes; signal/noise ratio, 3; signal intensity tolerance, 30% and minimum intensity, 100000. Peak intensities were subsequently normalized to the total spectral intensity and the normalized data were used to predict the molecular formula based on molecular ion peaks, additive ions and fragment ions. Next, the peaks were referenced to the mzCloud (www.mzcloud.org/), MassList and mzVault database and the accurate qualitative and relative quantitative results were generated. The statistical software R (R version R-3.4.3), Python (Python 2.7.6 version) and CentOS (CentOS release 6.6) were employed to perform statistical analyses.

### Metabolomics analysis

Identified metabolites were annotated using the databases as previously described [29]. We analyzed the metabolomics data via the website-based tool MetaboAnalyst (www.metaboanalyst.ca). Especially, among all analyses, we performed univariate analysis (t test) to generate the statistical significance (*P* value) and metabolites with variable importance in projection (VIP) > 1 and *P* value < 0.05 and fold change (FC) ≥ 1.5 or FC ≤ 0.67 were considered as differentially expressed.

### Microcomputed tomography (μCT) analysis

Dissected femur bones (on the other side that weren’t used for metabolite extraction) from C57BL6/J mice were fixed in 4% paraformaldehyde for 48 h and then were subjected to bone architecture analysis via μCT scanning with a Scanco vivaCT 40 instrument (Scanco Medical), as we described in our previous study [30]. Three-dimensional images were reconstructed via the built-in software at 10.5 μm resolution. The region of interest (ROI) in the trabecular bone started at 50 slices (525 μm) below the lowest point of the growth plate in distal femur and the range was 80 slices (840 μm). ROI in the cortical bone was chosen at the midshaft of femur and the range was 50 slices (525 μm). One constant threshold (184) was set for trabecular bone segmentation and the other (263) was set for cortical bone segmentation. Trabecular and cortical morphometry parameters including bone volume fraction (BV/TV), trabecular spacing (Tb.Sp), trabecular number (Tb.N), trabecular thickness (Tb.Th), cortical thickness (Ct.Th), cortical area (Ct.Ar), relative cortical-area-to-total-area ratio (Ct.Ar/Tt.Ar) and total area (Tt.Ar) were measured.

### Static and dynamic histomorphometry

For static histomorphometry, after fixed in 4% paraformaldehyde for 48h, femur bones were subsequently decalcified in 12% ethylenediaminetetraacetic acid (EDTA) solution for 3 weeks before being paraffin-embedded. 5-μm-thick sections were cut and stained by tartrate-resistant acid phosphatase (TRAP) staining and Masson’s trichrome (MT) staining according to standard methods. A kit (Sigma-Aldrich, 387A-1KT) was used for TRAP staining. We captured digital images of sections at 100× and 400× magnifications and took measurements 200 μm underneath growth plates and cortical bones. Multinucleated TRAP-positive cells lying on bone surface on TRAP-stained sections were identified as osteoclasts and red-stained rod-like or fish-like cells adhering to bone surface on MT-stained sections were identified as osteoblasts. Parameters including osteoclast surface (Oc.S/BS), osteoblast number (N.Ob/B.Pm) and osteoclast number (N.Oc/B.Pm) were calculated.

For dynamic histomorphometry, sterile filtered calcein (Sigma-Aldrich, C0875) was dissolved and formulated as a solution at 1 mg/mL in 2% NaHCO3 solution. Mice were double-labelled with calcein (20 mg/kg body weight) via intraperitoneal injection 9 days and 2 days before euthanasia. Then calcein-labelled femurs were fixed in ethanol and embedded in optimal cutting temperature (OCT) compound. 5-μm-thick undecalcified calcein-labelled sections were cut and visualized under a fluorescence microscope. We captured digital images of sections at 200× magnification and took measurements starting from 15% of femoral length proximal to distal epiphyseal growth plate and extending proximally for a total of 10% of femoral length. Parameters including total bone perimeter (B.Pm, mm), single- and double-labelled perimeter (sL.Pm and dL.Pm, mm) were measured. And mineral apposition rate (MAR = distance between double labels / 7 days, μm/day), mineralizing surface (MS/BS = [1/2 sL.Pm + dL.Pm] / B.Pm × 100, %) and bone formation rate (BFR/BS = MAR × MS/BS, μm^2^/μm/day) were calculated. Both static and dynamic parameters were calculated using Bioquant Osteo software (v18.2.6; Bioquant Image Analysis Corp.), and we applied the nomenclature recommended by American Society for Bone and Mineral Research [31].

### Immunostaining

For immunostaining, 5-μm-thick paraffin-embedded sections were dewaxed and rehydrated. Then endogenous peroxidase activity was blocked by 6% hydrogen peroxide. Next the slides were incubated with primary antibodies at 4 °C overnight. The primary antibodies included rabbit anti-osteocalcin (Abcam, ab93876), anti-b-galactosidase (Abcam, ab616) and anti-glutaminase (Abcam, ab156876). An HRP/DAB detection system purchased from Abcam was used for secondary antibody incubation and 3,3-diaminobenzidine (DAB) staining. Secondary antibody alone was used as the control. After color development via DAB staining, hematoxylin was used for nuclear staining. Digital images were captured at 200× or 400× magnification, senescent cells detected by b-galactosidase (b-gal) staining were analyzed by Image J software (National Institutes of Health, USA), and the number of OCN-positive cells per bone perimeter (N.OCN^+^/B.Pm) was calculated by Bioquant Osteo software.

### In vitro osteoclast differentiation, in vitro osteogenic differentiation and cell proliferation assay

BMM isolated from 8-week-old wildtype C57BL6/J male mice and BMSC isolated from 6-month-old and 18-month-old wildtype C57BL6/J male mice via above-mentioned methods were used for most in vitro experiments unless specified. For osteoclast differentiation, BMM were seeded in 96-well plates at 1.0×10^4^ cells/well with growth medium (α-MEM with 10% FBS and 30 ng/ml M-CSF) and on next day the medium was replaced by α-MEM containing 30 ng/ml M-CSF, 50 ng/ml RANKL and 10% FBS for an additional 5 days to induce osteoclast differentiation. TRAP staining was performed to identify TRAP-positive cells with 3 or more nuclei as osteoclasts via a kit (Sigma-Aldrich, 387A-1KT). For osteogenic differentiation, BMSC were seeded at 2.0×10^4^ cells/well in 12-well plates with growth medium (α-MEM with 10% FBS) and induced with osteogenic differentiation medium consisting of α-MEM, 10% FBS, 100 mg/ml ascorbic acid, 100mM dexamethasone and 10 mM b-glycerophosphate when reaching 80% confluency. Alizarin red staining was performed after 18 days of osteogenic differentiation and the quantification of alizarin red stain was completed via the method previously reported [32]: briefly, each well was washed in PBS for three times and then in 20% methanol/ 10% methanol/ 10% acetic acid, absorbance at 450 nm was measured via spectrophotometry.

Cell proliferation ability was assessed using CCK8 assay (Boster Biotechnology). BMM and BMSC were both seeded on 96-well plates at 3.0×10^3^ cells/well and cultured with growth medium respectively. After 1, 3, or 5 days, the assay was performed following manufacturer’s instructions and absorbance at 450 nm was measured with a microplate reader.

### Resorption activity assay of osteoclast

BMM were seeded in 6-well plates and induced with α-MEM containing 30 ng/ml M-CSF, 50 ng/ml RANKL and 10% FBS for 3 days. Then we digested the differentiating pre-osteoclasts with 0.25% trypsin and seeded equal number of cells on bone slices cultured cells with osteoclast differentiation medium containing M-CSF and RANKL until mature osteoclast formation confirmed by TRAP staining. Next, we incubated bone slices with 2 N sodium hydroxide and wiped away cells. The bone slices were then incubated with peroxidase-conjugated wheat germ agglutinin (Sigma, L3892) for 30 min and dyed by DAB (Abcam, ab64238) for 10 min. Images were captured at 100× magnification and 5 distinct areas of resorption pits in each bone slice were analyzed by image J software to calculate total bone resorption area and resorption area per osteoclast.

### Glutaminase (GLS) activity assay and extracellular oxygen consumption rate (OCR) assay

Both GLS activity and extracellular OCR of primary murine cells (BMSC or BMM) were measured via commercially available kits (ab284547 and ab197243 respectively) purchased from Abcam, following manufacturer’s instructions. Signals were recorded with a SpectraMax i3x microplate reader (Molecular Devices) at default parameters set by the operator and were plotted and analyzed by Graphpad PRISM 8.0 software (Graphpad Software Inc., USA).

**Figure 1.**
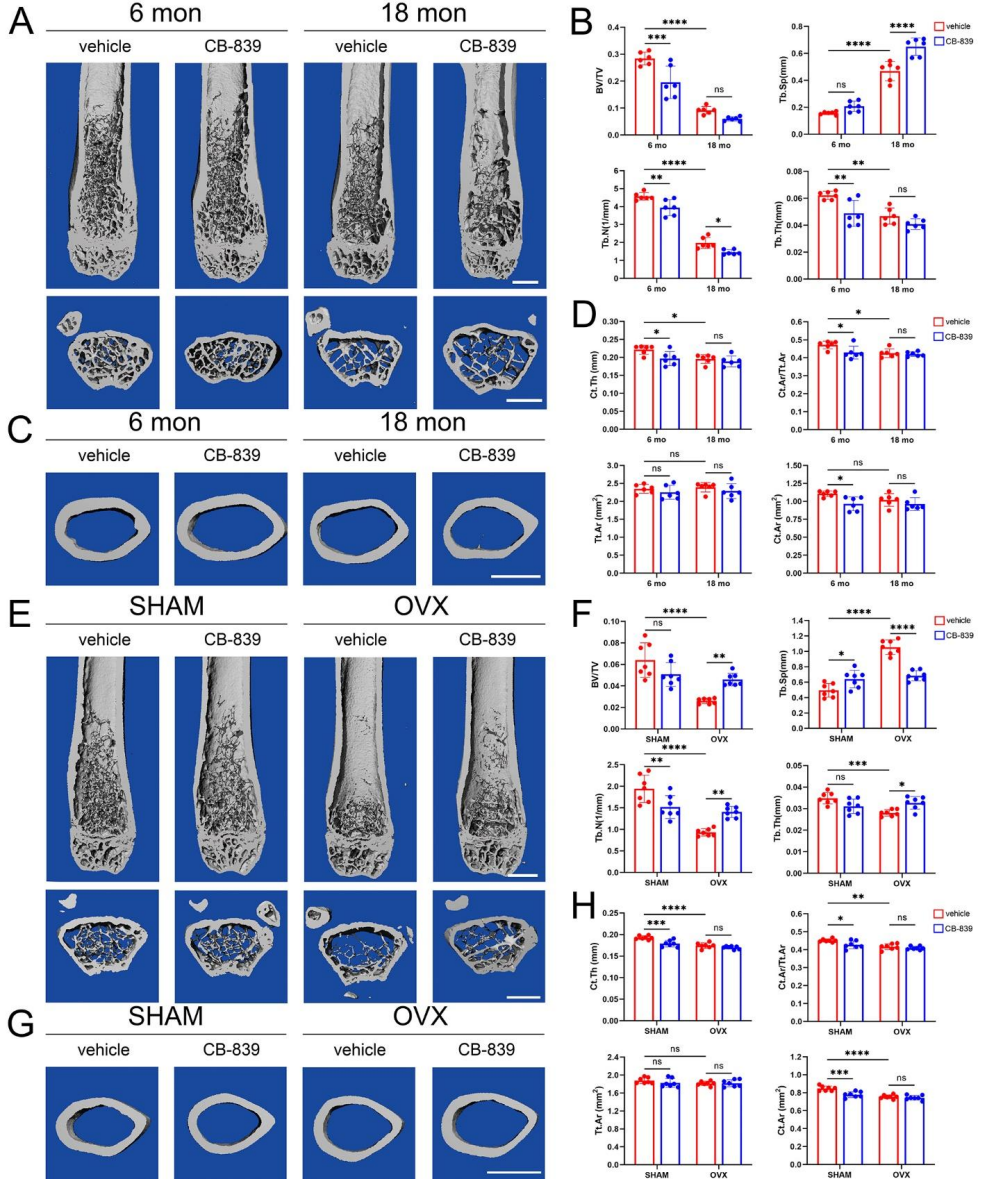
**Inhibiting GLS via CB-839 aggravates age-related bone loss in male mice while prevents OVX-induced bone loss in female mice**. (**A-D**) Distal femurs from 6-month-old and 18-month-old male groups treated with CB-839 (200 mg/kg body weight) or the same volume of vehicle twice daily were analyzed. Representative distal femoral μCT images (above is the longitudinal view, below is the axial view of metaphysis), scale bars represent 1 mm respectively (**A**); quantitative analysis of trabecular bone parameters including bone volume fraction (BV/TV), trabecular spacing (Tb.Sp), trabecular number (Tb.N) and trabecular thickness (Tb.Th), n = 6/group respectively (**B**); representative midshaft femoral μCT images (axial view), scale bar represents 1 mm (**C**); quantitative analysis of cortical bone parameters including cortical thickness (Ct.Th), cortical area (Ct.Ar), relative cortical-area-to-total-area ratio (Ct.Ar/Tt.Ar) and total area (Tt.Ar), n = 6/group respectively (**D**). (**E-H**) Distal femurs from sham-operated and ovariectomized groups treated with CB-839 (200mg/kg body weight) or same volume of vehicle twice daily were analyzed. Representative distal femoral μCT images (above is the longitudinal view, below is the axial view of metaphysis), scale bars represent 1 mm respectively (**E**); quantitative analysis of trabecular bone parameters including bone volume fraction (BV/TV), trabecular spacing (Tb.Sp), trabecular number (Tb.N) and trabecular thickness (Tb.Th), n = 7/group respectively (**F**); representative midshaft femoral μCT images (axial view), scale bar represents 1 mm (**G**); quantitative analysis of cortical bone parameters including cortical thickness (Ct.Th), cortical area (Ct.Ar), relative cortical-area-to-total-area ratio (Ct.Ar/Tt.Ar) and total area (Tt.Ar), n = 7/group respectively (**H**). Lines and error bars represent mean S.D.; all *P* values were determined by two-way ANOVA with Tukey’s multiple comparisons test (* *P* < 0.05, ** *P* < 0.01, *** *P* < 0.001, and **** *P* < 0.0001; “ns” not significant).

### Immunoblotting analysis

Total cell lysates were extracted from cultured cells and protein expressions were then determined following standard western blot procedures as previously described [10]. The following antibodies were used: anti-MPC1 (ab74871) and GLS1 (ab156876) from Abcam, anti-MPC2 (20049-1-AP) from Proteintech Group (Chicago, IL, USA) and anti-β-actin (BM0627) from Boster Biotechnology.

### Statistical analysis

We used GraphPad PRISM 8.0 software for statistical analyses except some involved in metabolite profiling and metabolomics analysis. All quantified data in histograms with scatter plots are presented as mean ± S.D. (standard deviation) and the normal distribution of data was examined by Shapiro-Wilk test. For normally distributed data, we applied the two-tailed unpaired Student’s t test for comparison between two groups and the one-way or two-way ANOVA followed by multiple comparisons test for comparison of multiple groups respectively if appropriate. For non-normally distributed data and data with very small sample size (n<5) as well, the Mann-Whitney test or the Kruskal-Wallis test followed by multiple comparisons test was used, as indicated in the figure legends. Every experiment was performed at least three times independently. For every result, a *P* value less than 0.05 was considered statistically significant and the representative image was presented.

## RESULTS

### Both inhibiting GLS via CB-839 and GLS1 knockdown via adenovirus prevent OVX-induced bone loss while aggravate age-related bone loss

To explore the effects of inhibiting GLS on different primary osteoporosis types in mice, we used CB-839, a potent and selective GLS inhibitor, to suppress GLS activity in vivo. We followed the dose, route and frequency of administration applied in a previous study which confirmed that oral administration of 200 mg/kg CB-839 twice daily could achieve peak concentrations of > 1 mmol/L or nmol/g in plasma or most mouse tissues except brain, and dramatically reduced GLS activity in these tissues [26]. Firstly, we did μCT analysis of distal femurs. Data revealed that both aging and OVX dramatically impaired microarchitecture and decreased trabecular bone mass in mice: significantly lower BV/TV, lower Tb.Th, lower Tb.N and higher Tb.Sp were detected both in the comparison between vehicle-treated 18-month-old mice and their 6-month-old sex-matched counterparts (Fig. 1A-B and Supplementary Fig. 1A-B) and in that between vehicle-treated ovariectomized mice and their sham-operated counterparts (Fig. 1E-F). Meanwhile, CB-839 treatment caused significant decreases in BV/TV, Tb.Th, and Tb.N in 6-month-old mice in both sexes and induced significantly decreased Tb.N and increased Tb.Sp in 18-month-old male mice and decreased Tb.Th and increased Tb.Sp in 18-month-old female mice, compared to vehicle treatment (Fig. 1A-B and Fig. 1A-B). Besides, although CB-839 treatment resulted in comparable Tb.Sp in young (6-month) mice, comparable BV/TV and Tb.Th in aged (18-month) male mice and comparable BV/TV and Tb.N in aged female mice, it decreased the mean values of BV/TV, Tb.N and Tb.Th and increased the mean values of Tb.Sp at above ages and sexes, compared to sex- and age-matched controls (Fig. 1A-B and Supplementary Fig. 1A-B). These results indicated that CB-839 treatment could not only cause trabecular bone loss in young mice, but also damage trabecular bone microstructure thus worsen age-associated osteoporosis in mice. However, CB-839 treatment in the same way as performed in 6-month-old and 18-month-old mice similarly resulted in significantly declined Tb.N, higher Tb.Sp and tendencies for BV/TV and Tb.Th to decrease with *P* values < 0.1 (p = 0.0936, p = 0.0703) in sham-operated mice while it led to greater BV/TV, greater Tb.N, greater Tb.Th and smaller Tb.Sp in ovariectomized mice, compared to their vehicle-treated controls (Fig. 1E-F). Unexpectedly, this part of results demonstrated the protective effects of CB-839 treatment on OVX-induced trabecular bone loss in mice. To verify the above effects of targeting GLS on trabecular bone, we intravenously injected adenovirus and confirmed GLS1 knockdown in bone tissue from male and female aged mice (Supplementary Fig. 2C, 2F), and also in that from ovariectomized mice (Supplementary Fig. 2I). Likewise, GLS1 knockdown caused remarkably reduced trabecular volume fraction, thickness, number and increased separation in aged mice (Supplementary Fig. 2A-B, D-E) while led to opposite results for trabecular bone parameters in ovariectomized mice (Supplementary Fig. 2G-H).

**Figure 2.**
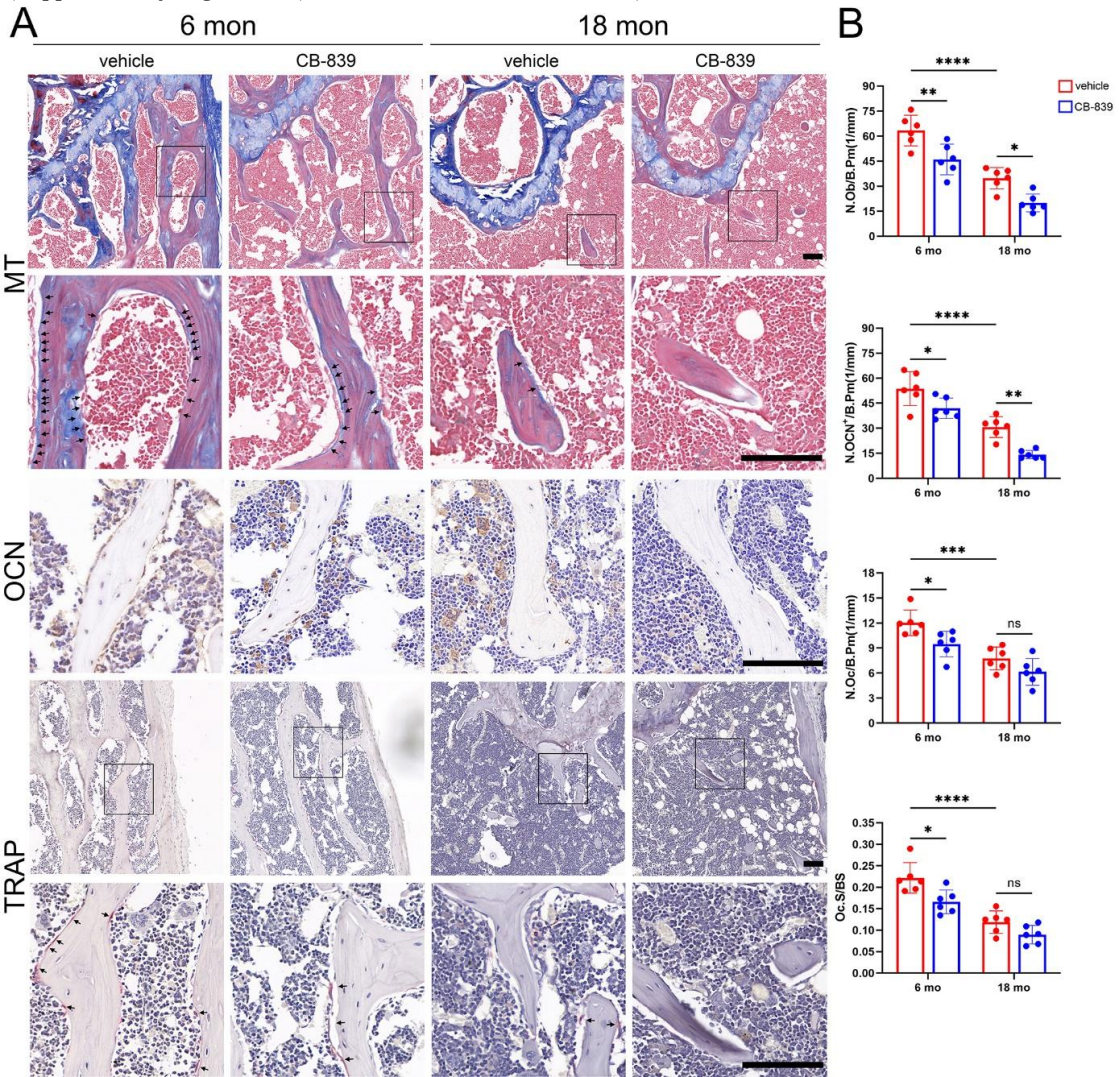
**CB-839 treatment alters bone turnover in the mouse model of age-related bone loss**. Distal femurs from 6-month-old and 18-month-old male groups treated with CB-839 (200 mg/kg body weight) or the same volume of vehicle twice daily were analyzed. (**A**) Representative metaphyseal region micrographs of sections stained with Masson’s trichrome (MT) (100× magnification above, 400× magnification below, osteoblasts labelled by black arrows), sections immunostained for osteocalcin (OCN) (400× magnification), and sections stained with tartrate-resistant acid phosphatase (TRAP) (100× magnification above, 400× magnification below, osteoclasts labelled by black arrows), scale bars represent 100 mm respectively. (**B**) Quantitative analysis of the number of osteoblasts per bone perimeter (N.Ob/B.Pm), the number of OCN-positive cells per bone perimeter (N.OCN^+^/B.Pm), the number of osteoclasts per bone perimeter (N.Oc/B.Pm) and the osteoclast surface fraction (Oc.S/BS), n = 6/group respectively. Lines and error bars represent mean S.D.; all *P* values were determined by two-way ANOVA with Tukey’s multiple comparisons test (* *P* < 0.05, ** *P* < 0.01, *** *P* < 0.001, and **** *P* < 0.0001; “ns” not significant).

**Figure 3.**
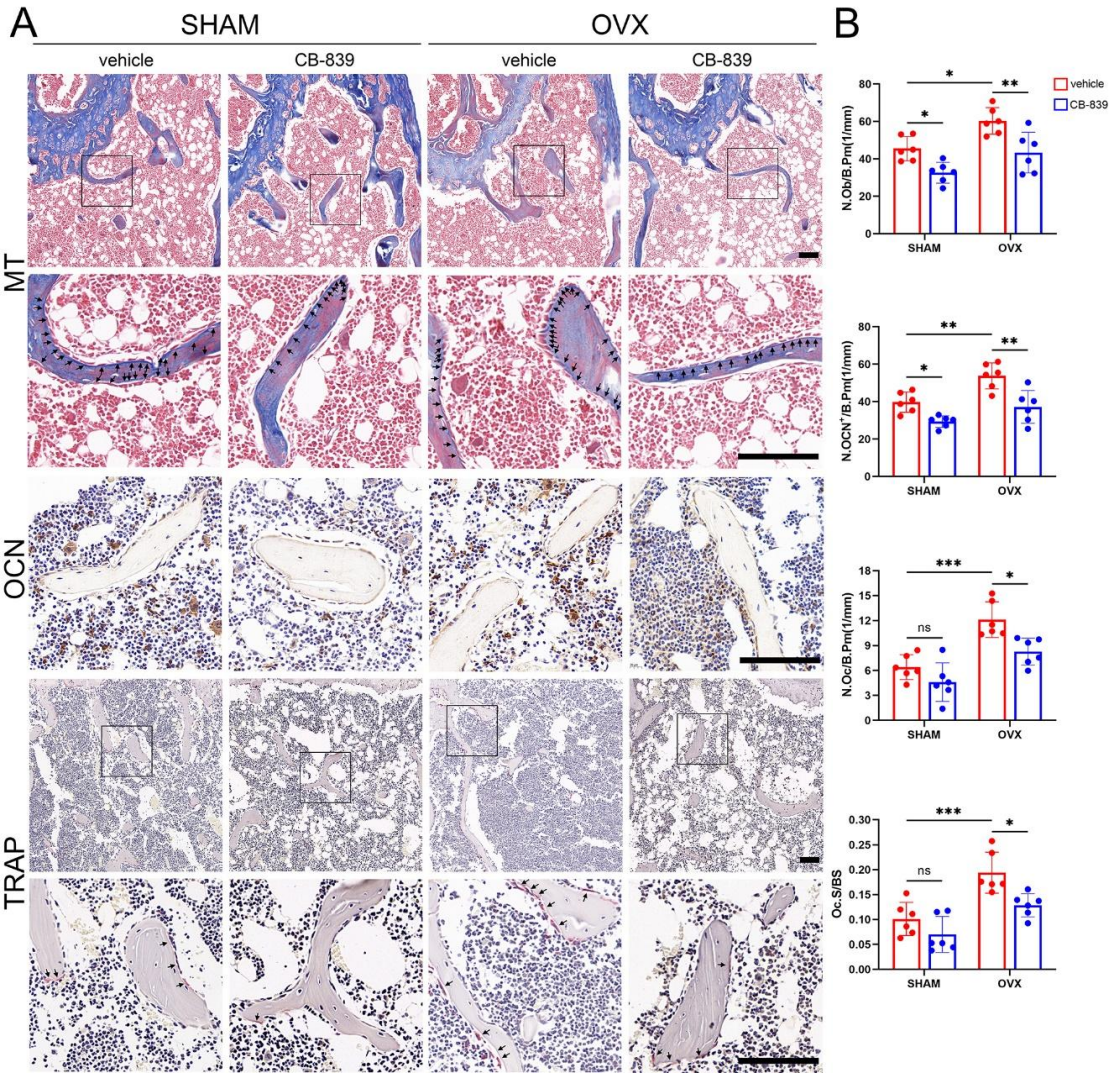
**CB-839 treatment alters bone turnover in the mouse model of OVX-induced bone loss**. Distal femurs from sham-operated and ovariectomized groups treated with CB-839 (200 mg/kg body weight) or the same volume of vehicle twice daily were analyzed. (**A**) Representative metaphyseal region micrographs of sections stained with Masson’s trichrome (MT) (100× magnification above, 400× magnification below, osteoblasts labelled by black arrows), sections immunostained for osteocalcin (OCN) (400× magnification), and sections stained with tartrate-resistant acid phosphatase (TRAP) (100× magnification above, 400× magnification below, osteoclasts labelled by black arrows), scale bars represent 100 mm respectively. (**B**) Quantitative analysis of the number of osteoblasts per bone perimeter (N.Ob/B.Pm), the number of OCN-positive cells per bone perimeter (N.OCN^+^/B.Pm), the number of osteoclasts per bone perimeter (N.Oc/B.Pm) and the osteoclast surface fraction (Oc.S/BS), n = 6/group respectively. Lines and error bars represent mean S.D.; all *P* values were determined by two-way ANOVA with Tukey’s multiple comparisons test (* *P* < 0.05, ** *P* < 0.01, *** *P* < 0.001, and **** *P* < 0.0001; “ns” not significant).

Next, we sought to analyze midshaft μCT data of femurs. It was noticed that aging and OVX also contributed to cortical bone loss: vehicle-treated aged (18-month) and ovariectomized mice both showed significantly lower Ct.Th, lower Ct.Ar/Tt.Ar and comparable Tt.Ar compared to their 6-month-old sex-matched and sham-operated counterparts respectively (Fig. 1C-D, G-H and Supplementary Fig. 1C-D). CB-839 treatment caused slightly but significantly reduced Ct.Th, Ct.Ar and Ct.Ar/Tt.Ar in 6-month-old mice while showed no distinguishable effect on above parameters in 18-month-old mice in both sexes, compared to vehicle-treated controls (Fig. 1C-D and Supplementary Fig. 1C-D). Similarly, CB-839 treatment was correlated with slightly declined Ct.Th, Ct.Ar, Ct.Ar/Tt.Ar and comparable Tt.Ar in sham-operated mice but induced no significant difference in these parameters in ovariectomized mice compared to vehicle treatment (Fig. 1G-H). These data revealed the impairments on cortical bone in young and sham-operated mice, with no evident impact on it in either aged or ovariectomized mice, by CB-839. In addition, GLS1 knockdown didn’t significantly alter any of the cortical bone parameters either in aged (Supplementary Fig. 2A-B, D-E) or in ovariectomized mice (Supplementary Fig. 2G-H). Therefore, μCT data suggested that both inhibiting GLS via CB-839 and GLS1 knockdown via adenovirus could accelerate the development of osteoporosis in aged mice but prevent that in ovariectomized mice, mainly in the aspect of trabecular bone mass.

**Figure 4.**
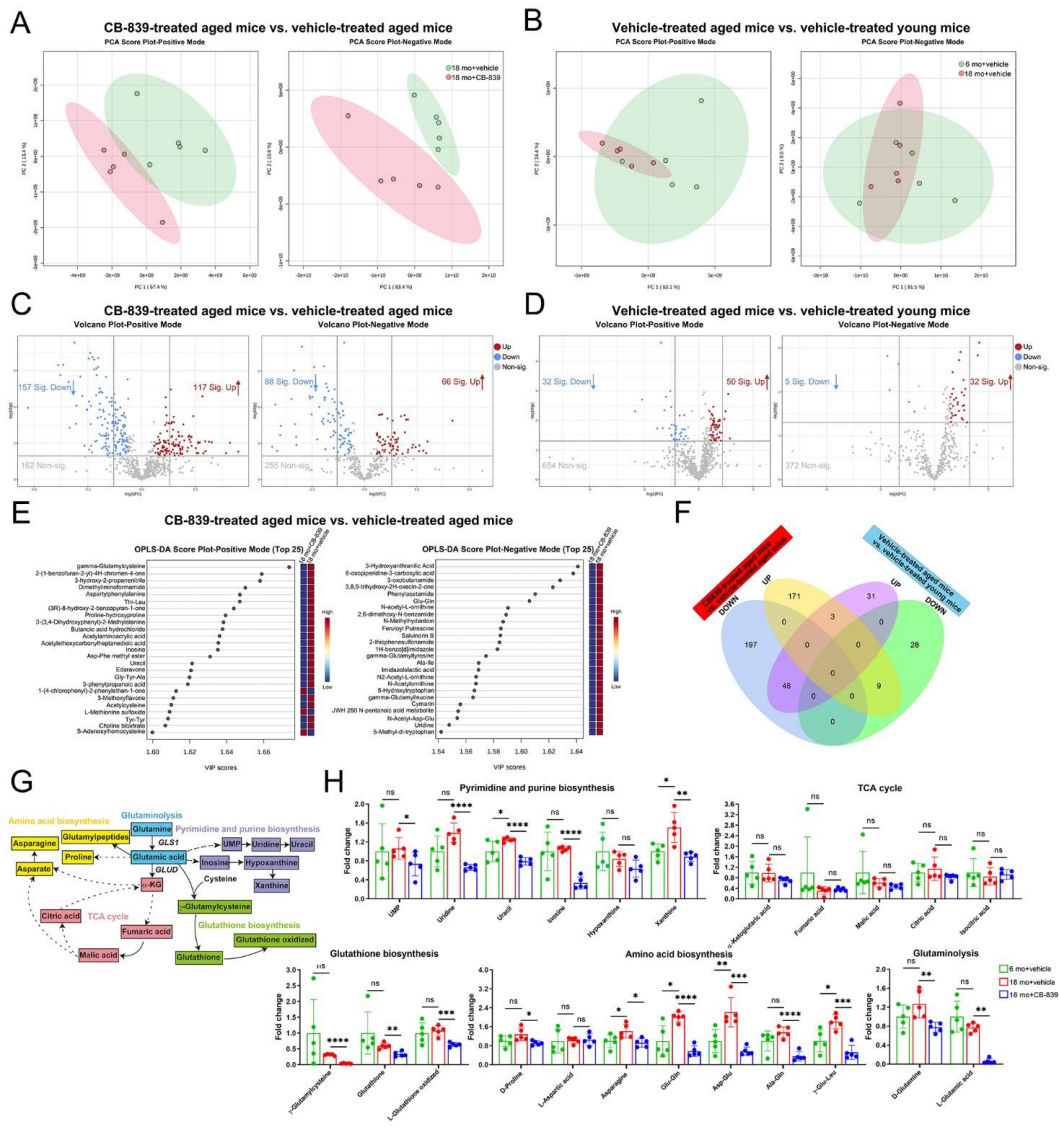
**CB-839 treatment alters the metabolic profile in BMSC from aged mice**. BMSC isolated from vehicle-treated mice at different ages (6-month and 18-month) and CB-839-treated aged (18-month) mice were subjected to metabolomics analysis, all data were analyzed and plotted by Metaboanalyst except the fold change plots in 4H. (**A-B**) Principal component analysis (PCA) plots for BMSC of CB839-treated and vehicle-treated aged mice (**A**), and for BMSC of vehicle-treated aged and young mice (**B**) in positive and negative modes, n = 5/group respectively. (**C-D**) Volcano plots for differentially expressed metabolites (VIP > 1 and *P* value < 0.05 and FC 1.5 or FC 0.67) between BMSC of CB-839-treated aged mice and that of vehicle-treated aged mice (**C**), and between BMSC of vehicle-treated aged mice and that of vehicle-treated young mice (**D**) in positive and negative modes, n = 5/group respectively. (**E**) Orthogonal partial least squares-discriminant analyses (orthoPLS-DA) for the most important, differentially expressed metabolites between CB-839-treated aged group and vehicle-treated aged group in positive and negative modes, n = 5/group. (**F**) Venn plot for the intersection between differentially expressed metabolites, no matter in positive or negative mode, in the comparison between CB-839-treated and vehicle-treated aged groups and that in the comparison between vehicle-treated aged and young groups, n = 5/group. (**G**) A diagram for main metabolic processes downstream of glutaminolysis mediated by glutaminase. (**H**) Fold change plots for stable intermediates involved in pyrimidine and purine biosynthesis, citric acid (TCA) cycle, glutathione biosynthesis, amino acid biosynthesis and glutaminolysis among three groups, n = 5/group respectively. In 4H, lines and error bars represent mean S.D., and the *P* values were determined by unpaired two-tailed Student’s t test (* *P* < 0.05, ** *P* < 0.01, *** *P* < 0.001, and **** *P* < 0.0001; “ns” not significant). Metabolomics data for analysis were shown in Supplementary Table 1.

## Effects of CB-839 treatment on bone mass are associated with alterations of bone turnover

To determine whether the effects of CB-839 treatment on above two types of osteoporosis were associated with alterations of bone turnover or not, we analyzed changes of bone resorption and formation by histomorphometric analyses on sections of distal femurs. Firstly, both osteoclastogenesis assessed by N.Oc/B.Pm and Oc.S/BS and osteoblastogenesis assessed by N.Ob/B.Pm and N.OCN^+^/B.Pm were dramatically reduced by aging while elevated by OVX (Fig. 2B and Fig. 3B). We found that CB-839 treatment significantly decreased N.Ob/B.Pm and N.OCN^+^/B.Pm at both 6-month-old and 18-month-old ages, while decreased N.Oc/B.Pm and Oc.S/BS only in 6-month-old mice but neither in 18-month-old mice, compared with vehicle-treated controls (Fig. 2A-B). On the other hand, CB-839 treatment significantly reduced N.Ob/B.Pm and N.OCN^+^/B.Pm in both sham-operated and ovariectomized mice, while reduced both N.Oc/B.Pm and Oc.S/BS only in ovariectomized mice but neither in sham-operated mice, compared with vehicle treatment (Fig. 3A-B). Furthermore, analysis of mineral apposition rate (MAR) and bone formation rate (BFR) uncovered that bone formation was largely suppressed by aging while was slightly but significantly activated by OVX (Supplementary Fig. 3A-D), in consistent with the former static histomorphometric results. Interestingly, CB-839 treatment significantly diminished MAR and BFR in both aged and ovariectomized mice (Supplementary Fig. 3B, 3D). Moreover, no significant difference in senescence-associated-β-galactosidase (SA-β-gal) positive cell number was detected between CB-839- and vehicle- treated aged mice (Supplementary Fig. 3A-B). These results implied that when bone turnover characterized by osteoclastogenesis, osteoblastogenesis and bone formation was in relatively active states such as young and ovariectomized mice, CB-839 treatment could suppress both bone formation and resorption; and when it was in relatively depressed states such as aged and sham-operated mice, this treatment could markedly suppress bone generation but barely affect bone resorption. And these suppressive effects of CB-839 on aged mice might have little correlation with cell senescence.

## CB-839 treatment alters metabolic profile in different orientations between BMSC of aged mice and BMM of ovariectomized mice

Firstly, in bone tissue immunoblotting and immunostaining analyses detected reduced GLS1 expression by aging while revealed elevated GLS1 expression by OVX (Supplementary Fig. 4A-E). Then enzyme activity analysis demonstrated that aging remarkably decreased while OVX increased intracellular GLS activity in bone marrow and confirmed efficient inhibition on GLS activity of bone marrow cells by CB-839 treatment in vivo (Supplementary Fig. 4F-G). These results indicated different basal skeletal glutaminase levels and activities between two mouse models and evident inhibitory effects on glutaminase by CB-839 treatment for both models.

Previous studies reported that decreased osteogenic differentiation accompanied with reduced self-renewal capacity of bone marrow mesenchymal stem/stromal cells (BMSC) accounted for impaired bone generation in age-related osteoporosis [13, 33, 34]. GLS activity of isolated BMSC was significantly decreased by aging and dramatically inhibited by CB-839 in vivo, consistent with that of total bone marrow cells (Supplementary Fig. 4H). For the reason that CB-839 treatment mainly hindered osteoblastogenesis and bone formation rather than osteoclastogenesis in aged mice, which was tightly associated with the impaired bone mass, we further subjected BMSC isolated from vehicle-treated mice at different ages and CB-839-treated aged mice to metabolomics analysis, to investigate the metabolic profile changes related with aging and GLS inhibition. The principal component analysis (PCA) plots showed much more distinct metabolite profiles between CB-839- and vehicle-treated 18-month-old groups than that between vehicle-treated 18-month-old and 6-month-old groups both in positive and negative modes (Fig. 4A-B). In accordance with this, volcano plots revealed that far more differentially expressed metabolites were in the comparison between CB-839-treated and vehicle-treated aged groups than that between vehicle-treated aged and young groups both in two modes (Fig. 4C-D). Next, we performed orthogonal partial least squares-discriminant analyses (orthoPLS-DA) to determine the most important, differentially expressed metabolites that contributed to metabolic pattern changes related to CB-839 treatment in aged murine BMSC and found that the highest ranked metabolites mainly belonged to amino acid metabolism, nucleotide metabolism, glutathione synthesis and cysteine metabolism, correlated with glutamate metabolism (Fig. 4E). The Venn plot also indicated that only a small part of metabolic pattern changes related to CB-839 treatment intersected with that related to aging (Fig. 4F). We thus deduced that CB-839 treatment rarely affected age-related metabolic alterations but its effects on aged murine BMSC were possibly lied on the aspect of glutaminolysis which predominately produced metabolites fueled for biosynthesis of amino acids, purines, pyrimidines and glutathione and in citric acid cycle (Fig. 4G). Based on this, we analyzed the fold changes of stable intermediates enrolled in the above processes among the groups. Consistent with the enzyme activity assay results, metabolite analysis also confirmed a sharp decrease in glutaminolysis caused by CB-839. Noticeably, CB-839 treatment significantly downregulated most of intermediates participating in biosynthesis processes of amino acids, purines, pyrimidines and glutathione, but barely affected citric acid cycle (Fig. 4H), suggesting that these processes were the primary influenced pathways when glutaminolysis was blocked in aged murine BMSC.

**Figure 5.**
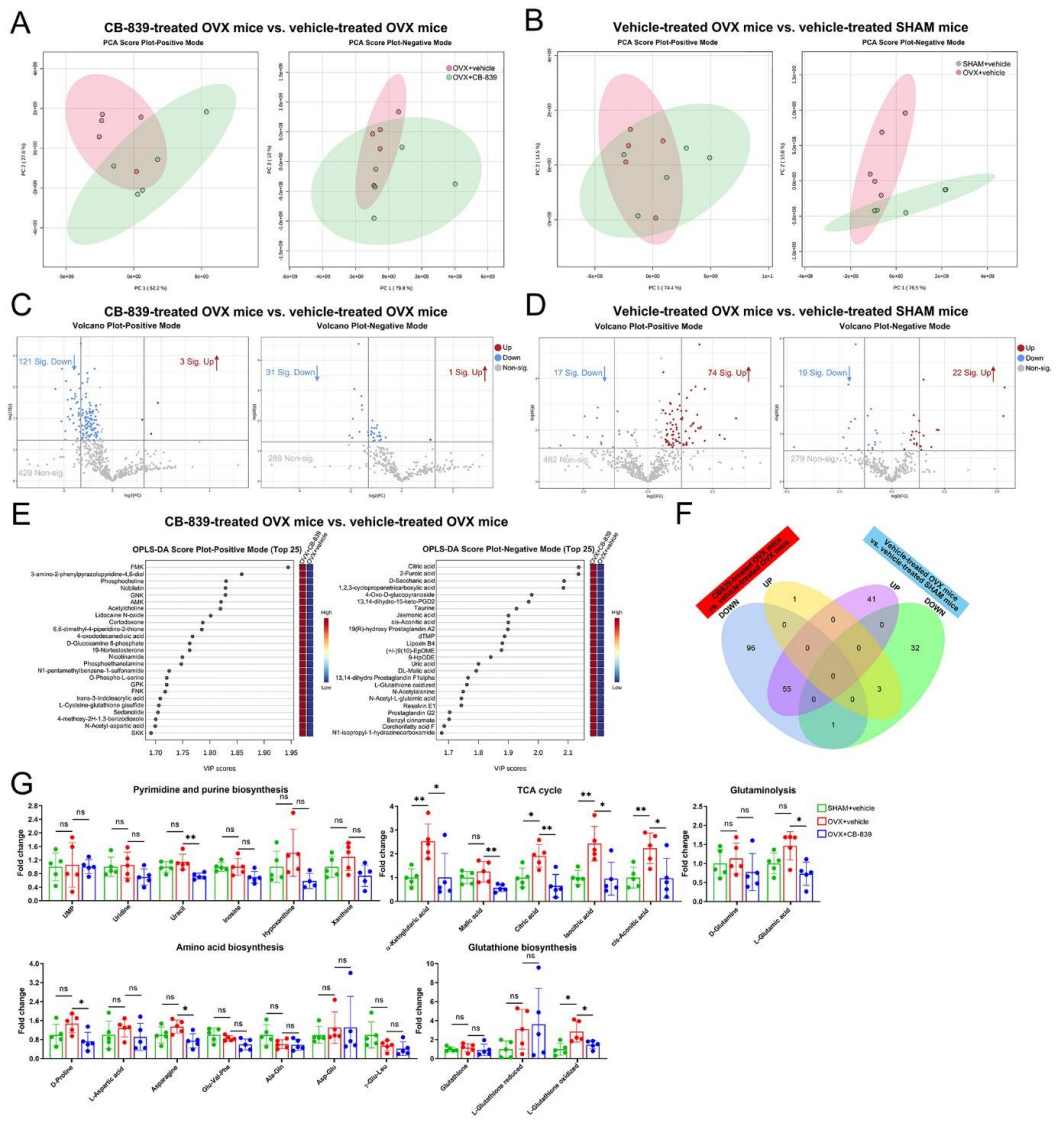
**CB-839 treatment alters the metabolic profile in BMM from ovariectomized mice**. BMM isolated from vehicle-treated sham-operated mice and CB-839- and vehicle-treated ovariectomized mice were subjected to metabolomics analysis, all data were analyzed and plotted by Metaboanalyst except the fold change plots in 5G. (**A-B**) Principal component analysis (PCA) plots for BMM of CB-839-treated and vehicle-treated ovariectomized mice (**A**), and for BMM of vehicle-treated sham-operated and ovariectomized mice (**B**) in positive and negative modes, n = 5/group respectively. (**C-D**) Volcano plots for differentially expressed metabolites (VIP > 1 and *P* value < 0.05 and FC 1.5 or FC 0.67) between BMM of CB-839-treated ovariectomized mice and that of vehicle-treated ovariectomized mice (**C**) and between BMM of vehicle-treated ovariectomized mice and that of vehicle-treated sham-operated mice (**D**) in positive and negative modes, n = 5/group respectively. (**E**) Orthogonal partial least squares-discriminant analyses (orthoPLS-DA) for the most important, differentially expressed metabolites between CB-839-treated ovariectomized group and vehicle-treated ovariectomized group in positive and negative modes, n = 5/group. (**F**) Venn plot for the intersection between differentially expressed metabolites, no matter in positive or negative mode, in the comparison between CB-839-treated and vehicle-treated ovariectomized groups and that in the comparison between vehicle-treated ovariectomized and sham-operated groups, n = 5/group. (**G**) Fold change plots for stable intermediates involved in pyrimidine and purine biosynthesis, citric acid (TCA) cycle, glutathione biosynthesis, amino acid biosynthesis and glutaminolysis among three groups, n = 5/group respectively. In 5G, lines and error bars represent mean S.D., and the *P* values were determined by unpaired two-tailed Student’s t test (* *P* < 0.05, ** *P* < 0.01, *** *P* < 0.001, and **** *P* < 0.0001; “ns” not significant). Metabolomics data for analysis were shown in Supplementary Table 2.

On the other hand, OVX greatly improved GLS activity of isolated BMM while CB-839 treatment significantly decreased that in ovariectomized mice, in accordance with that of total bone marrow cells (Supplementary Fig. 4I). CB-839 treatment resulted in not only impaired osteoblastogenesis and bone formation but also dampened osteoclastogenesis in ovariectomized mice and the latter should be the key factor responsible for decelerating OVX-induced bone loss correlated with overactivated osteoclasts. Hence, we subjected BMM isolated from vehicle-treated sham-operated mice and CB-839- and vehicle-treated ovariectomized mice to metabolomics analysis. Likewise, more distinct metabolic profiles with more differentially expressed metabolites related to the effects of CB-839 on BMM of ovariectomized mice than that related to the effects of OVX on BMM were detected in PCA and volcano plots respectively (Fig. 5A-D), and most of the altered metabolites related to the former factor didn’t intersect with that related to the latter as shown by the Venn plot (Fig. 5F). Unlike that in BMSC of aged mice, the top-ranked differentially expressed metabolites with highest VIP scores contributing to metabolic profile alterations related to CB-839 treatment in BMM of ovariectomized mice predominately belonged to citric-acid-cycle-related metabolism, amino acid metabolism, nucleotide metabolism and glutathione oxidation that correlated with glutamate metabolism, as displayed in orthoPLS-DA (Fig. 5E). Glutaminolysis was lessened by CB-839 as confirmed in metabolite analyses (Fig. 5G). Interestingly, fold change analyses of stable intermediates enrolled in processes downstream of glutaminolysis revealed that most of metabolites fueled in citric acid cycle, a fraction of metabolites fueled for purine and pyrimidine biosynthesis, and few metabolites fueled for amino acid biosynthesis were downregulated by CB-839, and glutathione oxidation was inhibited by CB-839 but not biosynthesis (Fig. 5G). We thus inferred that CB-839 treatment barely affected OVX-related metabolic alterations in BMM and the above-mentioned processes were the main affected pathways when glutaminolysis was blocked in BMM of ovariectomized mice.

### Different metabolic processes mediate glutaminase blockade effects between aging-impaired osteogenic differentiation and RANKL-induced osteoclast differentiation

Based on the above results of metabolomics analyses, we next aimed to prove the link between metabolic processes repressed by CB-839 and abnormal BMSC or BMM differentiation related to altered bone turnover via in vitro experiments. BMSC from male mice at different ages (6-month-old and 18-month-old) and BMM from 8-week-old male mice were isolated to achieve aging-impaired osteogenic differentiation and RANKL-induced osteoclast differentiation, for the study of decreased osteoblastogenesis and bone formation by aging and overactivated osteoclastogenesis by OVX respectively. Before drug treatments, immunoblotting analysis revealed different profiles but the same increasing trends of GLS1 expressions between differentiation processes of BMM and BMSC isolated from 8-week-old male mice (Supplementary Fig. 5A); IC50 values for glutaminase inhibition by CB-839 following preincubation for 24h with BMM, BMSC, osteoclasts (OC) differentiated from BMM and osteoblasts (OB) differentiated from BMSC were also distinct (Supplementary Fig. 5B). This part of data implied differences in GLS1 expressions and sensitivities to CB-839 among the bone-resorptive and bone-formative cells and their progenitors.

Then we performed in vitro studies with treatments. Firstly, after treatment with increasing concentrations at 1 to 4 mM for up to 5days, CB-839 showed an evident tendency to inhibit proliferation of aged murine BMSC in normal growth medium while didn’t affect it in osteogenic medium and displayed no significant effect on BMM proliferation in normal growth medium (Supplementary Fig. 5D, 5G). With the concentrations at 1 to 2 mM, CB-839 largely impaired GLS activity of both BMSC and BMM (Supplementary Fig. 5C, 5F), decreased osteogenic differentiation capacity of young and aged BMSC (Supplementary Fig. 5E) and suppressed RANKL-induced osteoclast differentiation and bone-resorption capacity of osteoclasts (Supplementary Fig. 5H, 5I) in a dose-dependent manner. Hence, these data demonstrated that inhibiting GLS via CB-839 resulted in abnormal functions of both BMSC and BMM in vitro.

**Figure 6.**
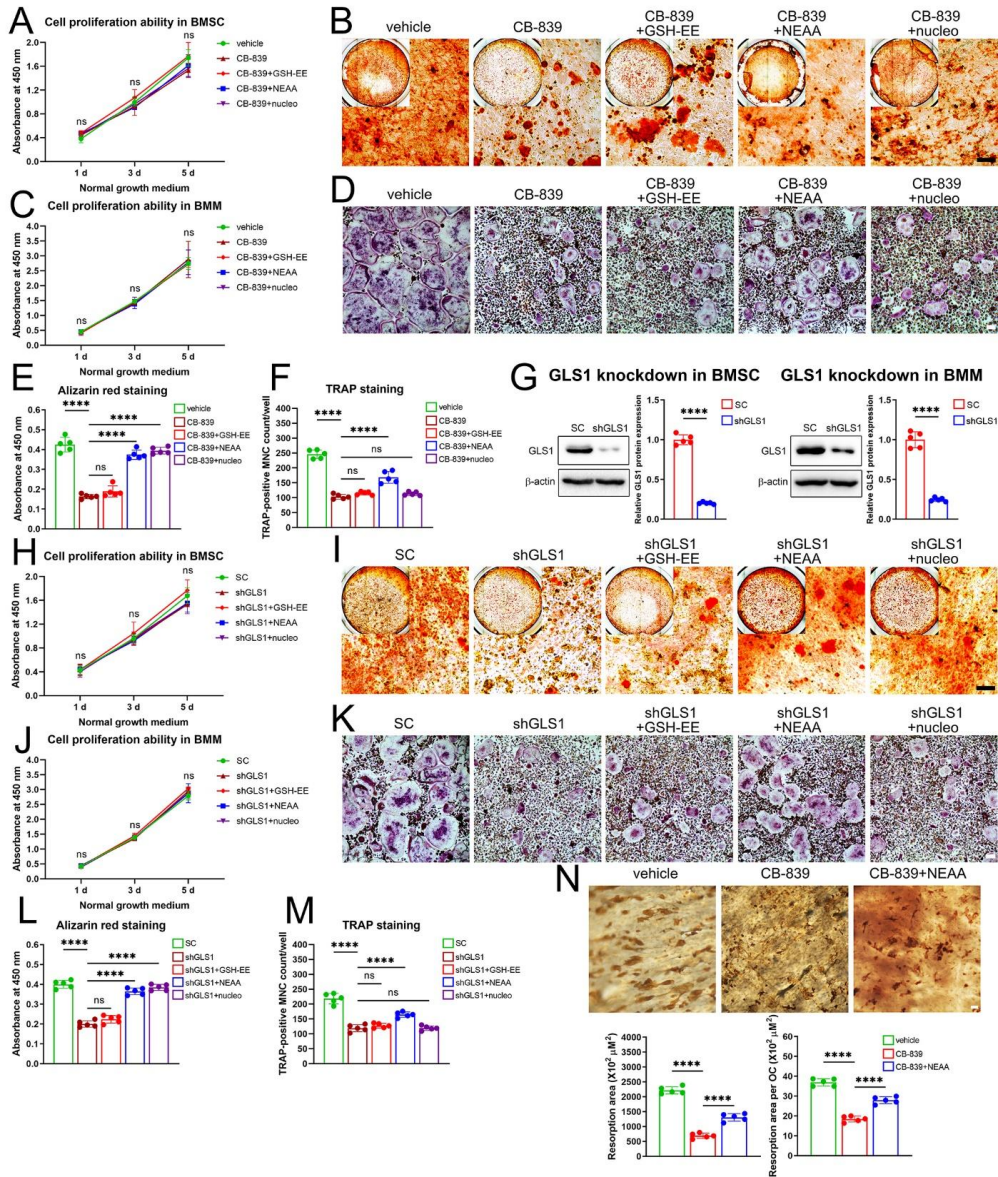
**Defective metabolic pathways upon GLS inhibition in aged BMSC play different roles between aging-impaired osteogenic differentiation and RANKL-induced osteoclast differentiation**. BMSC from aged (18-month-old) male mice and BMM from 8-week-old male mice were isolated. (**A-B**) BMSC were cultured in normal growth medium in presence of vehicle (DMSO), CB-839 (2 mM, the same below), CB-839 accompanied with GSH-EE (1 mM, the same below), CB-839 accompanied with 1× non-essential amino acids (NEAA), and CB-839 accompanied with 1× nucleotide mixture (nucleo) respectively, for up to 5 days for cell proliferation ability evaluation by CCK8 assay (n = 3/group) (**A**), or cultured in osteogenic differentiation medium in presence of these treatments for 18 days for alizarin red staining (representative micrographs are shown, scale bar represents 100 mm) (**B**). (**C-D**) BMM were cultured in normal growth medium in presence of the same treatments as that in 6A for up to 5 days for cell proliferation ability evaluation (n = 3/group) (**C**) or cultured with M-CSF and RANKL in presence of these treatments for 5 days for TRAP staining (representative micrographs are shown, scale bar represents 100 mm) (**D**). (**E-F**) Quantitative analysis of the alizarin red staining in 6B (**E**) and that of the number of osteoclasts in 6D (**F**), n = 5/group respectively. (**G**) GLS1 knockdown via adenovirus transduction of shRNAs (shGLS1) in BMSC and that in BMM were examined by western blots, representative images and quantitative analyses were shown, n=5/group respectively. (**H-I**) BMSC were treated with scrambled shRNAs (SC), shGLS1, shGLS1 accompanied with GSH-EE, shGLS1 accompanied with 1× NEAA, and shGLS1 accompanied with 1× nucleo respectively, cultured in normal growth medium for up to 5 days for cell proliferation ability evaluation (n = 3/group) (**H**), or cultured in osteogenic differentiation medium for 18 days for alizarin red staining (representative micrographs are shown, scale bar represents 100 mm) (**I**). (**J-K**) BMM with the same treatments as that in 6H were cultured in normal growth medium for up to 5 days for cell proliferation ability evaluation (n = 3/group) (**J**) or cultured with M-CSF and RANKL for 5 days for TRAP staining (representative micrographs are shown, scale bar represents 100 mm) (**K**). (**L-M**) Quantitative analysis of the alizarin red staining in 6I (**L**) and that of the number of osteoclasts in 6K (**M**), n = 5/group respectively. (**N**) BMM were firstly induced to differentiate into pre-osteoclasts and then digested and seeded on bone slices, treated with vehicle, CB-839, and CB-839 accompanied with 1× NEAA respectively, for osteoclastic resorption activity assay by DAB staining, representative micrographs are shown, and scale bar represents 100 mm; total bone resorption area and resorption area per osteoclast were calculated, n = 5/group respectively. Lines and error bars represent mean S.D.; *P* values in 6A, 6C, 6H, 6J were determined by Kruskal-Wallis test with Dunn’s multiple comparisons test, *P* values in 6G by unpaired two-tailed Student’s t test and the rest by one-way ANOVA with Sidak’s multiple comparisons test (* *P* < 0.05, ** *P* < 0.01, *** *P* < 0.001, and **** *P* < 0.0001; “ns” not significant).

Secondly, we investigated roles of the three primary defective metabolic pathways inside of aged murine BMSC upon GLS inhibition. Because inhibiting GLS displayed far more effects on osteogenic differentiation than proliferation capacity of aged murine BMSC especially upon osteogenic induction, we mainly paid attention to the former and used CB-839 at a concentration of 2 mM which revealed much less impacts on proliferation other than osteogenic differentiation (Supplementary Fig. 5D, 5E). Moreover, to validate the effects of CB-839 and exclude the off-target effects, we knocked down the expression of GLS1 in BMM and BMSC via adenovirus transduction of shRNAs (Fig. 6G). To target the critical metabolites decreased in the defective metabolic processes, we supplemented aged BMSC with a mixture of nucleotides (nucleo), non-essential amino acids (NEAA) and a cell-permeable glutathione ethyl ester (GSH-EE) respectively, which were validated not to affect BMSC proliferation up to 5 days in rescue experiments (Fig. 6A, 6H), and found that decreased osteogenic differentiation capacity of both aged BMSC treated with CB-839 and that with GLS1 knockdown could be largely restored by nucleotides and NEAA, but couldn’t be restored by GSH-EE (Fig. 6B, 6E, 6I, 6L). Meanwhile, we supplemented BMM with the same treatments that showed no harm to proliferation in rescue experiments (Fig. 6C, 6J), and detected that both the impaired osteoclastogenesis caused by CB-839 treatment and that related to GLS1 knockdown could be partially improved by NEAA but couldn’t be rescued by nucleotides or GSH-EE (Fig. 6D, 6F, 6K, 6M). Besides, NEAA also partially recovered resorptive osteoclast formation as confirmed by pit formation assays (Fig. 6N). Taken together, these data suggested the pivotal roles of amino acid biosynthesis and nucleotide biosynthesis in aged BMSC during osteogenic differentiation and the important role of the former in BMM during RANKL-induced osteoclast differentiation upon GLS inhibition.

**Figure 7.**
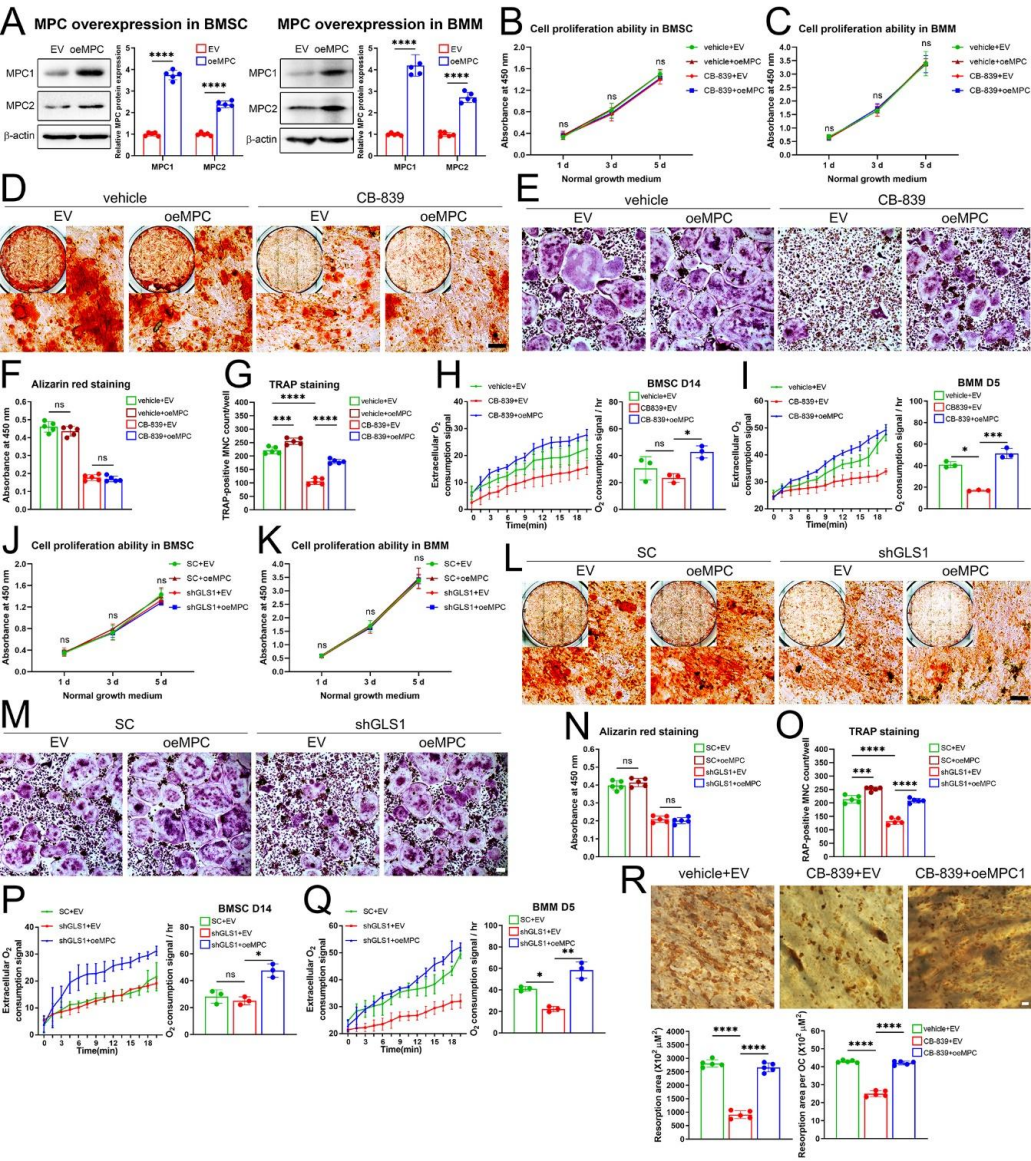
**Defective TCA cycle upon GLS inhibition in BMM plays different roles between aging-impaired osteogenic differentiation and RANKL-induced osteoclast differentiation**. BMSC from aged (18-month-old) male mice and BMM from 8-week-old male mice were isolated. (**A**) MPC overexpression via transduction of adenovirus overexpressing wildtype MPC1 and MPC2 (oeMPC) in BMSC and that in BMM were examined by western blots, representative images and quantitative analyses were shown, n = 5/group respectively. (**B-C**) BMSC treated with vehicle (DMSO) accompanied by empty vector (EV), vehicle accompanied by MPC overexpression, CB-839 (2 mM, the same below) accompanied by EV, and CB-839 accompanied by MPC overexpression respectively (**B**), or BMM with these treatments (**C**) were cultured in normal growth medium for up to 5 days for cell proliferation ability evaluation by CCK8 assay, n = 3/group respectively. (**D-E**) BMSC with the same treatments as that in 7B were cultured in osteogenic differentiation medium for 18 days for alizarin red staining (representative micrographs are shown, scale bar represents 100 mm) (**D**), BMM with the same treatments as that in 7C were cultured with M-CSF and RANKL for 5 days for TRAP staining (representative micrographs are shown, scale bar represents 100 mm) (**E**). (**F-G**) Quantitative analysis of alizarin red staining in 7D (**F**) and that of the number of osteoclasts in 7E (**G**), n = 5/group respectively. (**H-I**) BMSC treated with vehicle accompanied by EV, CB-839 accompanied by EV, and CB-839 accompanied by MPC overexpression respectively, were cultured in osteogenic differentiation medium for 14 days and then extracellular oxygen consumption rate (OCR) was assayed and calculated (**H**), BMM with these treatments were cultured with M-CSF and RANKL for 5 days and then OCR was assayed and calculated (**I**), n = 3/group respectively. (**J-K**) BMSC treated with scrambled shRNAs (SC) accompanied by EV, SC accompanied by MPC overexpression, shGLS1 accompanied by EV, and shGLS1 accompanied by MPC overexpression respectively (**J**), or BMM with these treatments (**K**) were cultured in normal growth medium for up to 5 days for cell proliferation ability evaluation, n = 3/group respectively. (**L-M**) BMSC with the same treatments as that in 7J were cultured in osteogenic differentiation medium for 18 days for alizarin red staining (representative micrographs are shown, scale bar represents 100 mm) (**L**), BMM with the same treatments as that in 7K were cultured with M-CSF and RANKL for 5 days for TRAP staining (representative micrographs are shown, scale bar represents 100 mm) (**M**). (**N-O**) Quantitative analysis of alizarin red staining in 7L (**N**) and that of the number of osteoclasts in 7M (**O**), n = 5/group respectively. (**P-Q**) BMSC treated with SC accompanied by EV, shGLS1 accompanied by EV, and shGLS1 accompanied by MPC overexpression respectively were cultured in osteogenic differentiation medium for 14 days and then extracellular oxygen consumption rate (OCR) was assayed and calculated (**P**), BMM with these treatments were cultured with M-CSF and RANKL for 5 days and then OCR was assayed and calculated, n = 3/group respectively (**Q**). (**R**) BMM were firstly induced to differentiate into pre-osteoclasts and then digested and seeded on bone slices, treated with vehicle accompanied by EV, CB-839 accompanied by EV, and CB-839 accompanied by MPC overexpression respectively, for osteoclastic resorption activity assay by DAB staining, representative micrographs are shown and scale bar represents 100 mm; total bone resorption area and resorption area per osteoclast were calculated, n = 5/group respectively. Lines and error bars represent mean S.D.; *P* values in 7B, 7C, 7H, 7I, 7J, 7K, 7P, 7Q were determined by Kruskal-Wallis test with Dunn’s multiple comparisons test, *P* values in 7A by unpaired two-tailed Student’s t test and the rest by one-way ANOVA with Sidak’s multiple comparisons test (* *P* < 0.05, ** *P* < 0.01, *** *P* < 0.001, and **** *P* < 0.0001; “ns” not significant).

Finally, we focused on the citric acid cycle in which metabolites involved were extensively downregulated in ovariectomized murine BMM but not in aged murine BMSC upon GLS inhibition. Mitochondrial pyruvate carrier (MPC), composed of MPC1 and MPC2 subunits, has been identified as the key transporter responsible for pyruvate entering the mitochondria, and overexpression of both subunits were proved to enhanced pyruvate influxes that guaranteed provision of citric acid cycle intermediates in several mammal cell types [35-37]. So, we achieved MPC overexpression both in aged BMSC and BMM via adenovirus transduction, the overexpressed protein levels were confirmed by western blots (Fig. 7A). Of note, MPC overexpression accompanied with CB-839 (2 mM) and GLS1 knockdown respectively didn’t alter proliferation capacity up to 5 days in both cell types (Fig. 7B-C, J-K) and MPC overexpression barely induce any changes to impaired osteogenic differentiation capacity of aged BMSC caused by CB-839 or GLS1 knockdown (Fig. 7D, 7F, 7L, 7N). In contrast, MPC overexpression greatly improved the suppressed osteoclastogenesis caused by CB-839 and that caused by GLS1 knockdown (Fig. 7E, 7G, 7M, 7O). Besides, MPC overexpression also largely restored osteoclastic bone resorption when GLS was suppressed (Fig. 7N). Interestingly, OCR assays at the 14th day of osteogenic induction revealed nearly no alteration resulted from CB-839 or GLS1 knockdown, and significant increases related to MPC overexpression in rescue experiments (Fig. 7H, 7P); while those at the 5th day of RANKL induction revealed significant decreases caused by CB-839 and GLS1 knockdown respectively, both of which could be dramatically rescued by MPC overexpression (Fig. 7I, 7Q). Accordingly, we implied that GLS blockade reduced oxidative phosphorylation in BMM but not in aged BMSC, consistent with the metabolomics data in the aspect of citric acid cycle intermediates (Fig. 4H, 5G), and increasing pyruvate influxes into mitochondria via MPC overexpression could greatly reverse the inhibitory effects of GLS blockade on osteoclastogenesis but could barely alter the impaired osteogenic differentiation capacity caused by that. Together, these data suggested the pivotal role of mitochondrial oxidative metabolism via citric acid cycle during RANKL-induced osteoclast differentiation upon GLS inhibition.

## DISCUSSION

Primary osteoporosis can be classified into two types according to its feature of bone remodeling: one occurs soon after menopause in women characterized by rapid trabecular bone loss (menopause-related osteoporosis), the other occurs in elder people including men and women characterized by slower loss of both cortical and trabecular compartments (age-related osteoporosis) [38, 39]. The pathogenic mechanism differs between these two types. For the former one, estrogen deficiency that increases bone turnover, enhances osteoclastic function while impairs compensatory osteoblastic function has been clearly clarified as the seminal mechanism [40, 41]; for the latter one, cellular senescence that decreases both new bone generation via osteoblasts and osteoclast-mediated bone resorption is the main cause [13, 42]. Correlated with these differences in cellular mechanism, monotherapy via anti-resorptive medications is always considered as the first option for postmenopausal osteoporosis treatment, while anabolic agents are recommended as an initial option in cases of aging patients at high risks of osteoporotic fracture whose bone turnover markers are at extremely low levels [43, 44]. In addition, sex steroid deficiency and cellular senescence played independent roles in the pathogenesis of osteoporosis [45]. Hence, it’s important to characterize the therapeutic roles and related mechanisms of a drug target in two types of primary osteoporosis. GLS1 is the critical enzyme responsible for glutamine catabolism and is essential for proliferation and osteogenic differentiation of osteoprogenitors [9, 10]. On the other hand, overexpression of GLS1 has been reported to accelerate osteoclast differentiation and either deprivation of glutamine or pharmacological inhibition of its transport dampens osteoclast differentiation and function in vitro [11, 12]. Therefore, GLS1 is regarded as a potential target for osteoporosis that may affect both bone resorption and formation. Nevertheless, the exact mechanism accounting for its role during osteoclast differentiation has never been elucidated and the effects of targeting GLS on primary osteoporosis and related bone turnover are not clear.

In this study, we built age-related bone loss and OVX-induced bone loss mouse models, and targeted GLS via the specified CB-839 treatment that proved to achieve peak concentrations of > 1mmol/L (or nmol/g) in most mouse tissues and efficiently reduce GLS activity [26], or genetic knockdown. In these ways, we showed that age-related bone loss was aggravated while the other was alleviated when GLS was inhibited (Fig. 1, S1-S2). Although GLS is widely expressed in different bone cell types as previously reported [46], many bone cell types such as osteocytes, sense the mechanical changes and mainly affect bone turnover by regulating bone-resorbing and bone-forming cells [47, 48]. Therefore, to explore the underlying mechanism mediating the effects of GLS inhibition on bone mass, this study only focused on the direct effectors responsible for bone remodeling and their progenitors where GLS activity was possibly tightly correlated with cell functions [9-11]. One step further, we found that the effects of GLS inhibition on bone turnover depended on situations: when bone remodeling was activated by OVX, CB-839 treatment predominately suppressed osteoclastogenesis and bone resorption; and when it was repressed by aging, the same treatment mainly impaired osteoblastogenesis and bone formation (Fig. 2-3, Supplementary Fig. 3). Given that aging and OVX exerted opposite impacts, both on bone tissue GLS expression and on GLS activity of bone marrow cells (Supplementary Fig. 4), we then speculated that these bone mass phenotypes caused by CB-839 treatment were correlated with the different effects of aging and OVX on bone remodeling.

**Figure 8.**
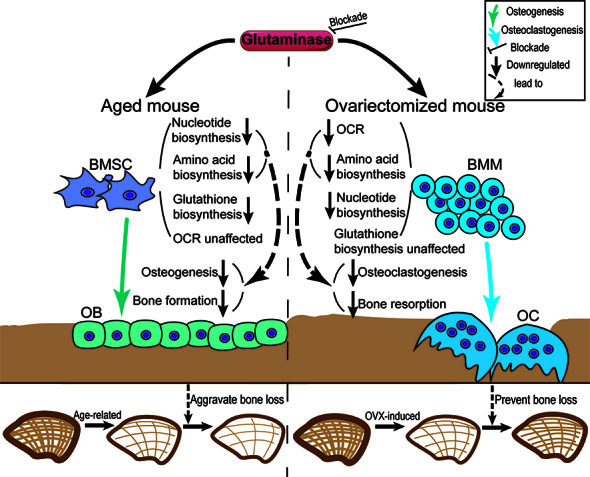
A graphic abstract of this article.

In age-related bone loss model, not only osteoclastogenesis but also osteoblastogenesis and bone formation were significantly suppressed by CB-839 at 6-month-old age, contributing to the murine phenotype of decreased trabecular bone mass and reduced cortical bone thickness (Fig. 1 and Fig. 2, Supplementary Fig. 1 and 3). Interestingly, CB-839 significantly suppressed osteoblastogenesis and bone formation other than osteoclastogenesis, while causing defects mainly in trabecular bone mass in aged mice (Fig. 1 and 2, Supplementary Fig. 1 and 3). Hence these suggested that CB-839 demonstrated much more suppressive effects on bone formation than resorption both in young and aged mice. Consistently, we observed much more GLS1 expression and sensitivity to CB-839 treatment in bone-formative cells than that in bone-resorptive cells isolated from the same donor, so were their progenitors, from the in vitro experiments (Supplementary Fig. 5). These could partially account for the more suppressive effects of CB-839 on bone formation than on bone resorption at different ages. Moreover, as shown in recent researches, despite impaired regeneration of both hematopoietic and skeletal stem cell (SSC) lineages with aging, aged SSC/BMSC promote myeloid bias and osteoclastogenesis in hematopoietic lineage, making bone resorption exceed formation, which is tightly connected to age-related metabolic changes inside of SSC/BMSC and is a driving cause of osteoporosis [49, 50]. We thus speculated that the effects of CB-839 treatment on bone remodeling and bone mass in aged mice were on one hand correlated with it predominate impairments on osteoblastogenesis and bone formation in vivo (Fig. 2, Supplementary Fig. 3), and on the other hand its nonsignificant effects on bone marrow SA-b-gel-positive cells (Supplementary Fig. 3) and on age-related metabolic profile alterations of BMSC (Fig. 4) might also provide evidences for its limited impact on senescent hSSC/BMSC-induced osteoclastogenesis.

Nevertheless, in OVX-induced bone loss model, CB-839 mainly decreased osteoblastogenesis and bone formation in sham-operated mice and resulted in damaged trabecular bone microstructure and cortical bone loss, while significantly decreased osteoclastogenesis, osteoblastogenesis and bone formation at the same time in ovariectomized mice, preventing trabecular bone loss without affecting cortical bone mass (Fig. 1 and 3, Supplementary Fig. 3). The direct effects of estrogen deficiency on bone formation are suppressive, however, its role in activating osteoclasts induces compensatory activation of osteogenesis, leading to activation of bone remodeling while creating a huge gap between bone resorption and formation [51]. In addition, in consideration of the elevated GLS1 expression and sensitivity to CB-839 during osteoclastogenesis (Supplementary Fig. 5), overactivation of osteoclasts by OVX could increase their sensitivity to CB-839 treatment at the same time. Accordingly, we deduced that in such a situation, the same CB-839 treatment as that in sham-operated mice exerted more inhibition on overactivated osteoclastogenesis and bone resorption than that on compensatory increased osteoblastogenesis and bone formation, and thus the gap was filled. The significantly increased osteoclastogenesis than osteoblastogenesis (Fig. 3) and dramatically elevated GLS activity in BMM in ovariectomized mice (Supplementary Fig. 4) provide evidences for this theory.

Importantly, we then uncovered that metabolic profiles were altered by CB-839 in different directions between aged murine BMSC and ovariectomized murine BMM (Fig. 4-5). In line with a previous study which investigated osteoprogenitors from GLS1-deficient 4-week-old male mice [10], our research proved the pivotal roles of generation of nucleotide and amino acids through GLS-mediated glutamine catabolism during osteogenic differentiation of aged murine BMSC, with similar rescue experiment designs (Fig. 6). Moreover, for the first time we illustrated that GLS-mediated fluxes to amino acids and TCA cycle (tricarboxylic acid cycle or citric acid cycle) intermediates were essential for RANKL-induced osteoclast differentiation of BMM, especially the latter (Fig. 7).

Recent studies revealed that glutamine-derived carbons were allocated to different metabolic fate to support cellular function in different skeletal cell types [10, 52]. However, in these researches, only progenitor cells from young mice were used in vitro. Consistent with these findings, our data indicate that glutamine catabolism is essential for osteogenic differentiation of aged murine BMSC mainly through biosynthesis of amino acids and nucleotides (Fig. 4 and 6). Different from the previous finding [10], GSH-EE didn’t significantly alter proliferation in aged BMSC, suggesting that aged BMSC required less de novo GSH synthesis to maintain cell viability (Fig. 6). On the other hand, our research showed that GLS-mediated carbon flux into TCA cycle could account for most of the effects of GLS blockade on RANKL-induced osteoclast differentiation (Fig. 5 and 7). But GLS blockade didn’t alter TCA cycle intermediates and OCR, neither did aged BMSC require GLS-mediated flux into TCA cycle during osteogenic differentiation as demonstrated in rescue experiments (Fig. 4 and 7). It’s known that energy required for osteoclast differentiation mainly derives from oxidative phosphorylation while glycolysis is regarded as the major metabolic pathway to meet energy demand during osteoblast differentiation [53, 54], and besides, glutamine and glucose are the two major sources of carbon fueled into TCA cycle and compensate for each other to maintain oxidative phosphorylation [55]. Based on these theories, our results imply that energy deficit caused by blocking glutamine catabolism during osteogenic differentiation of aged BMSC may be easily compensated by other metabolism process such as glycolysis, while glutamine catabolism during RANKL-induced osteoclast differentiation is far more essential and the deficiency of it cannot be rescued by increasing carbon flux from other energy sources. Therefore, blocking GLS in aged BMSC and young BMM resulted in decreased osteogenic differentiation and osteoblast differentiation via biosynthetic and bioenergetic deficits respectively.

Taken together, we studied bone mass phenotypes and bone remodeling characteristics related to CB-839 treatment in mouse models via both in vivo and in vitro experiments. Consequently, our research demonstrated that the different outcomes between two types of osteoporosis in mice caused by inhibiting GLS via CB-839 were tightly connected to the suppressive effects on both aging-impaired osteoblastogenesis and OVX-enhanced osteoclastogenesis mediated by different metabolic processes downstream of glutaminolysis. Previous studies have proposed that glutaminolysis could supply energy and metabolic intermediates necessary for physiological bone remodeling [9, 10, 12]. Here, our study uncovered the effects of targeting GLS on the progression of primary osteoporosis and provided evidence for glutaminolysis inhibition as a possible strategy for preventing menopause-related osteoporosis but a path should be avoided while treating age-related osteoporosis. There are some limits in our study: firstly, we didn’t use gene knock-out mice or different doses of drug other than the one proved to inhibit GLS in the former study in the animal studies; secondly, we just applied histomorphological studies on sections but didn’t perform studies on cell lineages related to bone resorption and formation; thirdly, lack of clinical studies and patient data restricted the implications of this study. Therefore, further research is still needed in the future.

## Supplementary Materials

The Supplementary data can be found online at: www.aginganddisease.org/EN/10.14336/AD.2024.0201.



## Data Availability

The authors declare that data supporting the findings of this study are available within the paper and its Supplementary Information files. The metabolomics datasets for Supplementary Table 1 and Supplementary Table 2 have been deposited in MetaboLights [56], and are accessible through the study identifier MTBLS9415 (https://www.ebi.ac.uk/metabolights/MTBLS9415).
