# Supplementary material for: Inhibiting Glutaminase Exerts Opposite Effects on Ovariectomy-Induced and Age-Related Reductions in Murine Bone Mass
Source: Aging Dis. 2024 Feb 1;16(1):432–53. doi: 10.14336/AD.2024.0201 (PMC11745427; doi:10.14336/AD.2024.0201)
Supplement: Supplementary file 1 [file AD-16-1-432-s.pdf]

SUPPLEMENTARY DATA

Inhibiting Glutaminase Exerts Opposite Effects on Ovariectomy-Induced and Age-Related Reductions in Murine Bone Mass

Qian Guo, Hongjian Zhao, Zijian Dong, Haozhe Cheng, Meipeng Zhu, Zhong Fang

© 2024. Guo Q et al. Published online at http://www.aginganddisease.org/EN/10.14336/AD.2024.0201

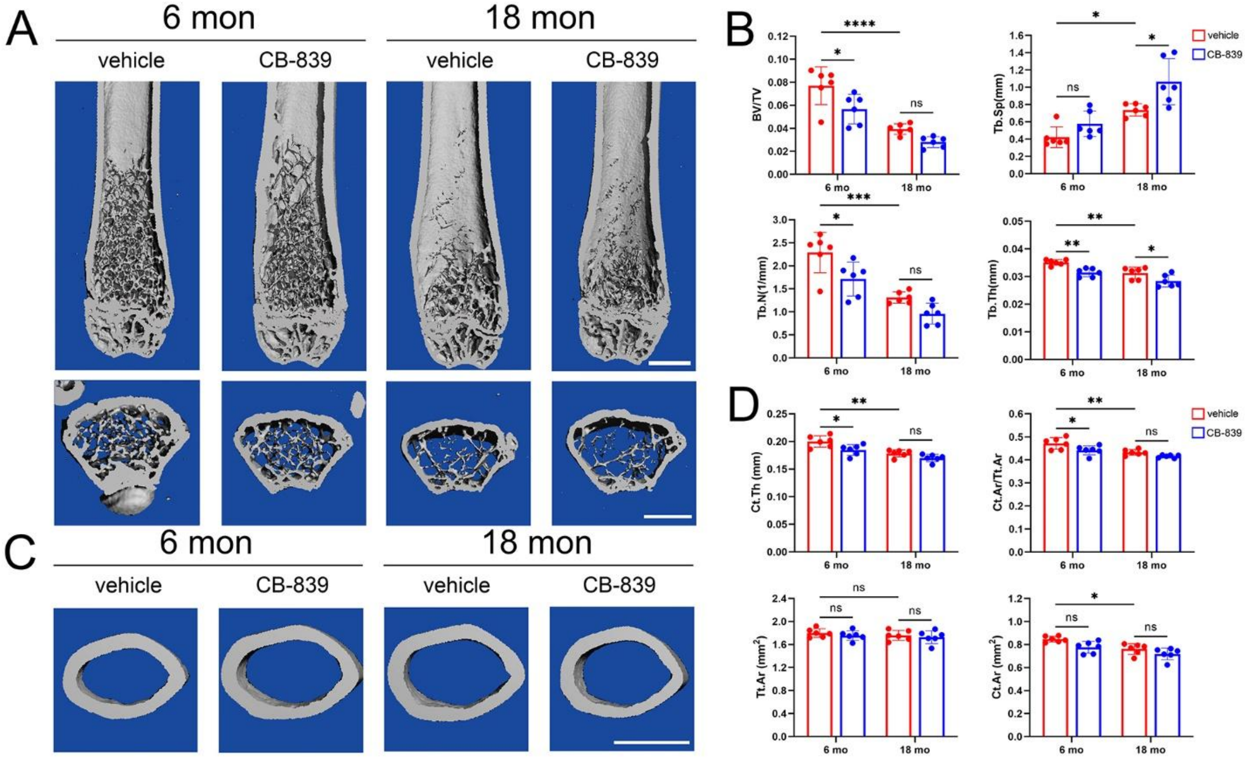

Supplementary Figure 1. Inhibiting GLS via CB-839 aggravates age-related bone loss in female mice. Distal femurs from 6-month-old and 18-month-old female groups treated with CB-839 (200 mg/kg body weight) or same volume of vehicle twice daily were analyzed. (A) Representative distal femoral μCT images (above is the longitudinal view, below is the axial view of metaphysis), scale bars represent 1 mm respectively. (B) Quantitative analysis of trabecular bone parameters including bone volume fraction (BV/TV), trabecular spacing (Tb.Sp), trabecular number (Tb.N), and trabecular thickness (Tb.Th), n = 6/group respectively. (C) Representative midshaft femoral μCT images (axial view), scale bar represents 1mm. (D) Quantitative analysis of cortical bone parameters including cortical thickness (Ct.Th), cortical area (Ct.Ar), relative cortical-area-to-total-area ratio (Ct.Ar/Tt.Ar) and total area (Tt.Ar), n = 6/group respectively. Lines and error bars represent mean ± S.D.; all *P* values were determined by two-way ANOVA with Tukey's multiple comparisons test (* $P < 0.05$, ** $P < 0.01$, *** $P < 0.001$, and **** $P < 0.0001$; "ns" not significant).

© 2024. Guo Q et al. Published online at http://www.aginganddisease.org/EN/10.14336/AD.2024.0201

SUPPLEMENTARY DATA

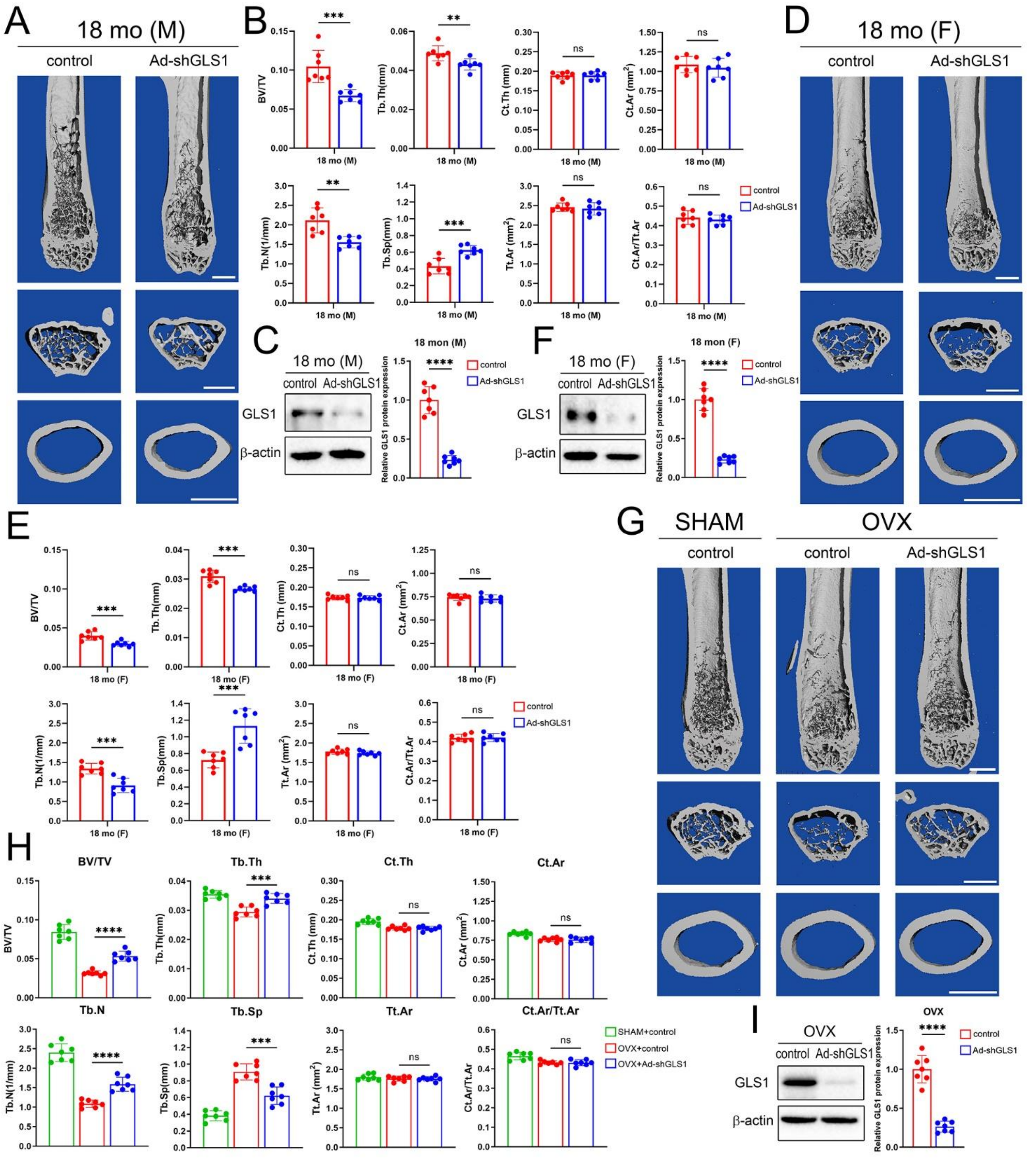

Supplementary Figure 2. GLS1 knockdown via adenovirus exacerbates age-related bone loss but improves OVX-induced bone loss in mice. (A-C) Distal femurs from 6-month-old and 18-month-old male groups weekly treated with adenovirus expressing shGLS1 or scrambled shRNAs (control) were analyzed. Representative distal femoral (above is the longitudinal view, middle is the axial view of metaphysis) and midshaft femoral (below, axial view) μCT images, scale bars represent 1 mm respectively (A); quantitative analysis of trabecular bone parameters including bone volume fraction (BV/TV), trabecular spacing (Tb.Sp), trabecular number (Tb.N) and trabecular thickness (Tb.Th), and cortical bone parameters including cortical thickness (Ct.Th), cortical area (Ct.Ar), relative cortical-area-to-total-area ratio (Ct.Ar/Tt.Ar) and total area (Tt.Ar), n = 7/group respectively (B); relative protein expressions of GLS1 in bone tissue were quantified by western blots, representative images and quantitative analysis were shown, n = 7/group (C). (D-F) Distal femurs from 6-month-old and 18-month-old female groups weekly treated with adenovirus expressing shGLS1 or scrambled shRNAs were analyzed. Representative distal femoral (above is the longitudinal view, middle is the axial view of metaphysis) and midshaft

femoral (below, axial view) μCT images, scale bars represent 1 mm respectively (D); quantitative analysis of trabecular bone parameters including bone volume fraction (BV/TV), trabecular spacing (Tb.Sp), trabecular number (Tb.N) and trabecular thickness (Tb.Th), and cortical bone parameters including cortical thickness (Ct.Th), cortical area (Ct.Ar), relative cortical-area-to-total-area ratio (Ct.Ar/Tt.Ar) and total area (Tt.Ar), n = 7/group respectively (E); relative protein expressions of GLS1 in bone tissue were quantified by western blots, representative images and quantitative analysis were shown, n = 7/group (F). (G-I) Distal femurs from sham-operated and ovariectomized groups weekly treated with adenovirus expressing shGLS1 or scrambled shRNAs were analyzed. Representative distal femoral (above is the longitudinal view, middle is the axial view of metaphysis) and midshaft femoral (below, axial view) μCT images, scale bars represent 1 mm respectively (G); quantitative analysis of trabecular bone parameters including bone volume fraction (BV/TV), trabecular spacing (Tb.Sp), trabecular number (Tb.N) and trabecular thickness (Tb.Th), and cortical bone parameters including cortical thickness (Ct.Th), cortical area (Ct.Ar), relative cortical-area-to-total-area ratio (Ct.Ar/Tt.Ar) and total area (Tt.Ar), n = 7/group respectively (H); relative protein expressions of GLS1 in bone tissue were quantified by western blots, representative images and quantitative analysis were shown, n = 7/group (I). Lines and error bars represent mean ± S.D.; all P values were determined by two-way ANOVA with Tukey's multiple comparisons test (* $P < 0.05$, ** $P < 0.01$, *** $P < 0.001$, and **** $P < 0.0001$; "ns" not significant).

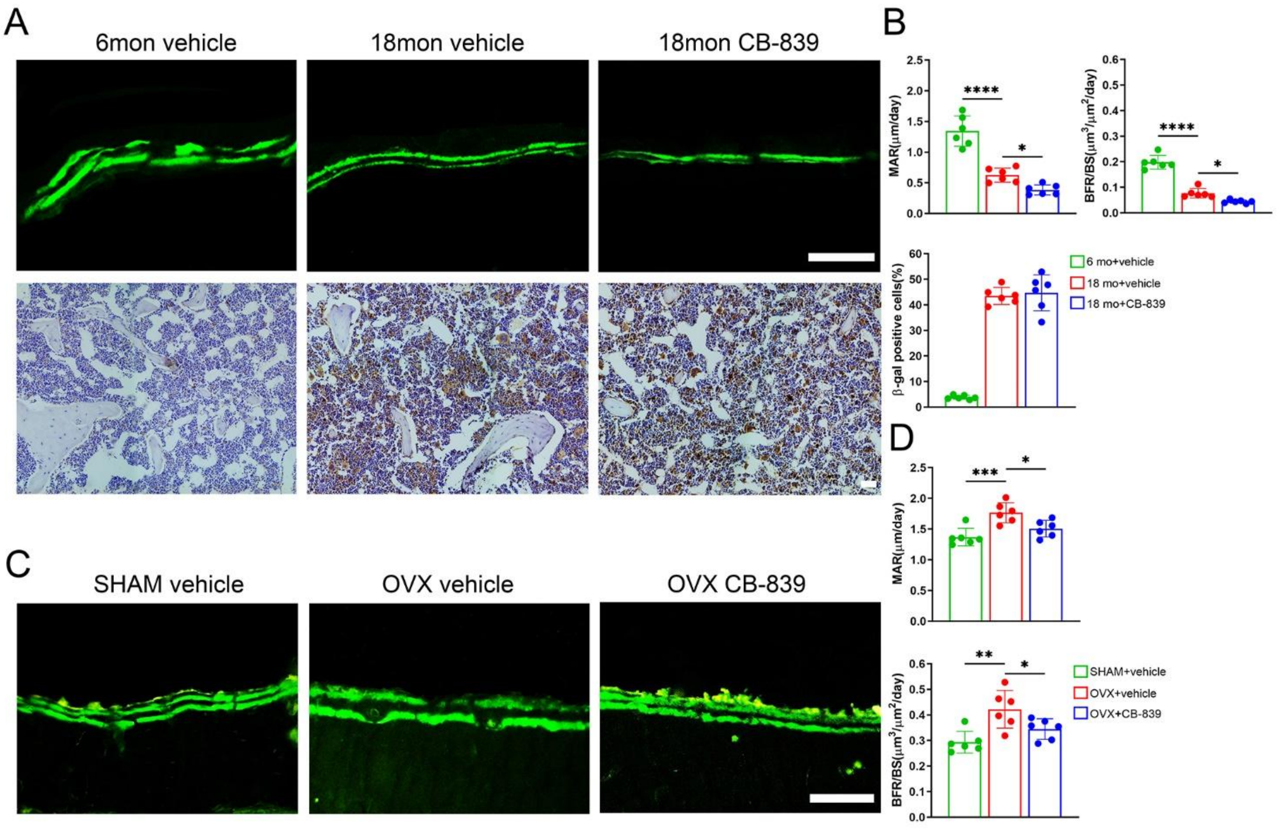

Supplementary Figure 3. CB-839 treatment alters bone formation in both two mouse models without affecting senescence-associated-b-galactosidase (SA-β-gal) positive cell count. (A-B) Distal femurs from 6-month-old and 18-month-old vehicle-treated, and 18-month-old CB-839-treated (200 mg/kg body weight) male groups were analyzed. Representative micrographs of calcein-labelled (above, 800× magnification) and SA-β-gal-stained sections (below, 200× magnification), scale bars represent 50 mm respectively (A); quantitative analysis of mineral apposition rate (MAR), bone formation rate (BFR/BS), and the percent of SA-β-gal-positive cells, n = 6/group respectively (B). (C-D) Distal femurs from sham-operated and ovariectomized vehicle-treated, and ovariectomized CB-839-treated (200 mg/kg body weight) groups were analyzed. Representative micrographs of calcein-labelled sections (800× magnification), scale bar represents 50 mm (C); quantitative analysis of mineral apposition rate (MAR) and bone formation rate (BFR/BS), n = 6/group respectively (D). Lines and error bars represent mean ± S.D.; all P values were determined by one-way ANOVA with Sidak's multiple comparisons test (* $P < 0.05$, ** $P < 0.01$, *** $P < 0.001$, and **** $P < 0.0001$; "ns" not significant).

© 2024. Guo Q et al. Published online at http://www.aginganddisease.org/EN/10.14336/AD.2024.0201

SUPPLEMENTARY DATA

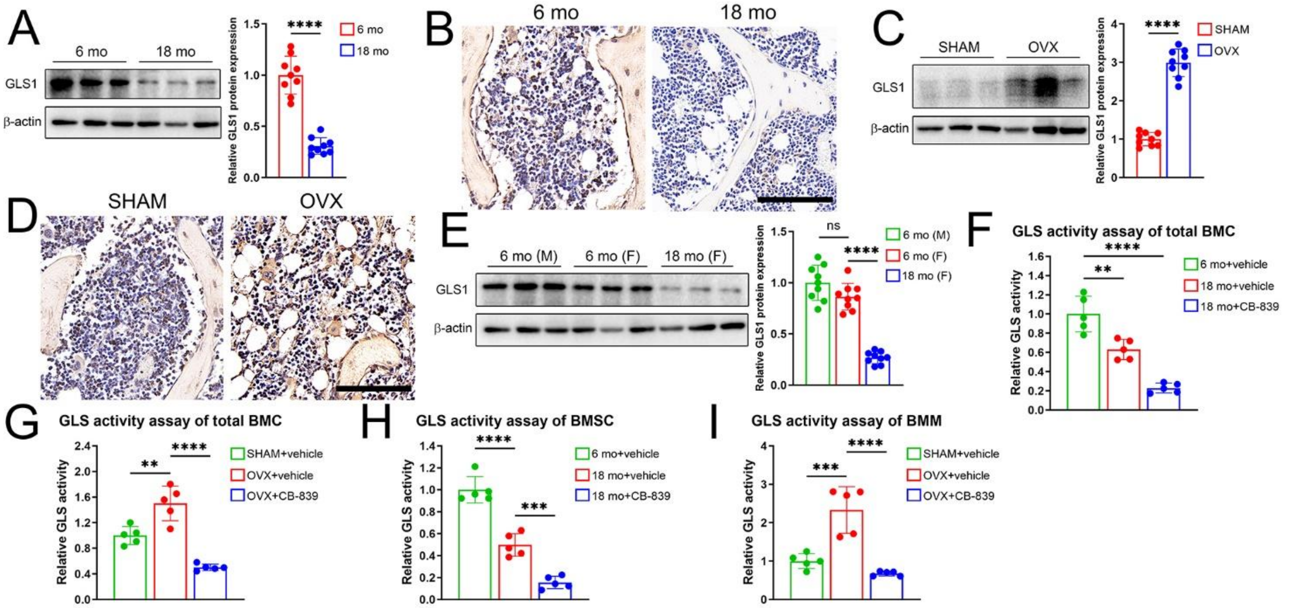

Supplementary Figure 4. Analysis of basal GLS levels between different mouse models and genders, and GLS activity assay results. (A-B) For male mice with different ages (6-month and 18-month): relative protein expressions of GLS1 in bone tissue were examined by western blots, representative images and quantitative analysis were shown, n = 9/group (A); representative metaphyseal region micrographs of sections immunostained for GLS1 (400× magnification), scale bar represents 100 mm (B). (C-D) For sham-operated and ovariectomized mice (treated with surgery at 10-week-old and sacrificed at 20-week-old): relative protein expressions of GLS1 in bone tissue were examined by western blots, representative images and quantitative analysis were shown, n = 9/group (C); representative metaphyseal region micrographs of sections immunostained for GLS1 (400× magnification), scale bar represents 100 mm (D). (E) Relative protein expressions of GLS1 in bone tissue, for 6-month male, 6-month female and 18-month female mice, were examined by western blots, representative images and quantitative analysis were shown, n = 9/group. (F-I) Quantitative analysis of GLS activity assay for total bone marrow cells (BMC) isolated from vehicle-treated mice at different ages (6-month and 18-month) and CB-839-treated aged (18-month) mice (F), for total BMC isolated from vehicle-treated sham-operated mice and CB-839- and vehicle-treated ovariectomized mice (G), for BMSC isolated from vehicle-treated mice at different ages (6-month and 18-month) and CB-839-treated aged (18-month) mice (H), and for BMM isolated from vehicle-treated sham-operated mice and CB-839- and vehicle-treated ovariectomized mice (I). Lines and error bars represent mean ± S.D.; *P* values in S4A, S4C were determined by unpaired two-tailed Student’s t test and the rest by one-way ANOVA with Sidak’s multiple comparisons test (* $P < 0.05$, ** $P < 0.01$, *** $P < 0.001$, and **** $P < 0.0001$; “ns” not significant).

© 2024. Guo Q et al. Published online at http://www.aginganddisease.org/EN/10.14336/AD.2024.0201

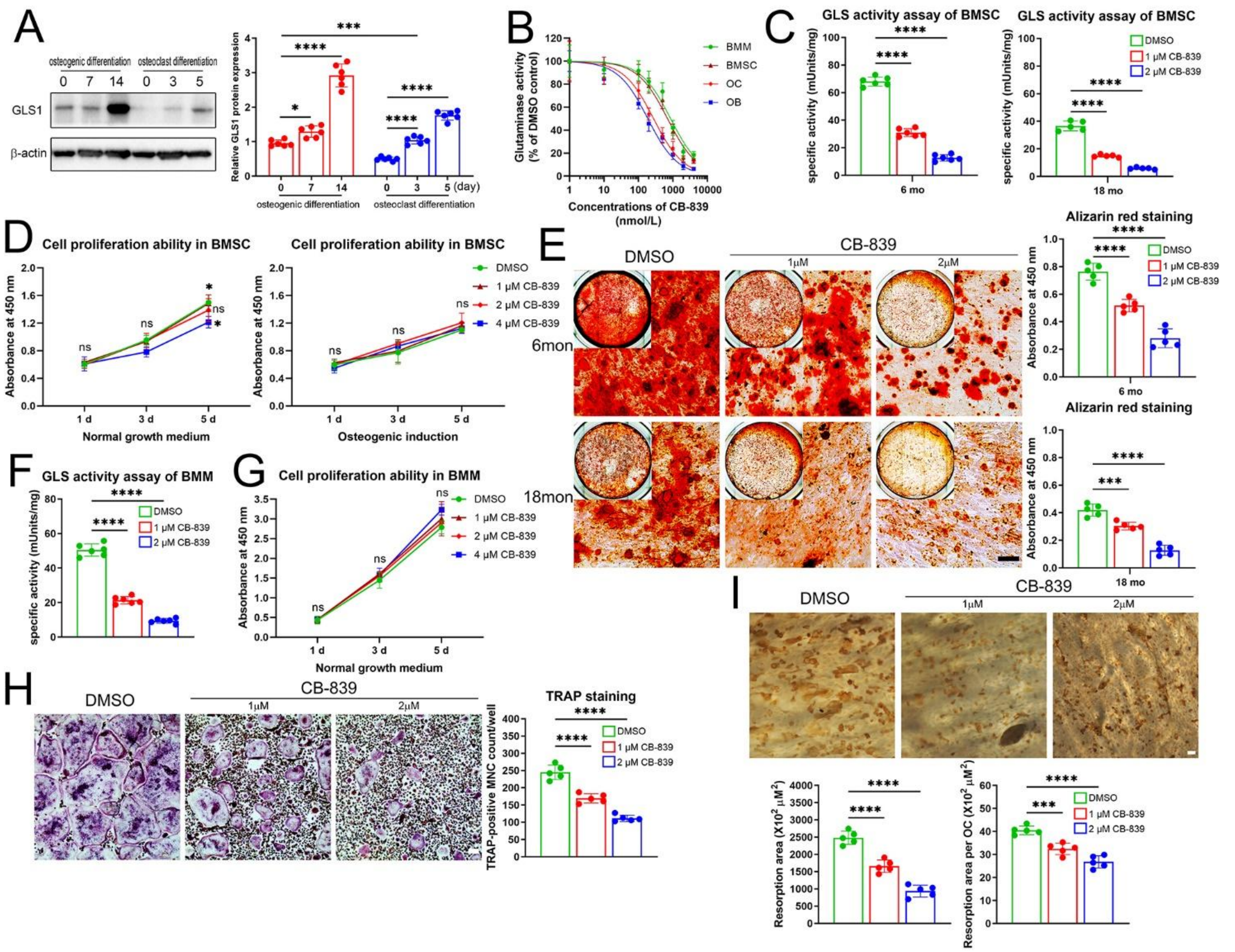

Supplementary Figure 5. Inhibiting GLS via CB-839 suppresses both aging-impaired osteogenic differentiation and RANKL-induced osteoclast differentiation. (A) BMSC and BMM isolated from 8-week-old male mice were cultured in osteogenic and osteoclastic differentiation mediums respectively for indicated days, relative cellular protein expressions of GLS1 were examined by western blots, representative images and quantitative analysis were shown, n = 6/group. (B) BMSC, BMM isolated from 8-week-old male mice, and osteoblasts (OB), osteoclasts (OC) differentiated from these cells respectively were preincubated with different concentrations of CB-839 for 24 h, and then dose-response curves were plotted to derive IC50 values in vitro, n = 3/group for every dose. (C) Young (6-month-old) or aged (18-month-old) murine BMSC were pretreated with DMSO and different concentrations of CB-839 (1, 2 mM) respectively for 24 h, and then subjected to GLS activity assay, n = 6/group respectively. (D) aged murine BMSC were cultured in the presence of DMSO and different concentrations of CB-839 (1, 2, 4 mM) respectively, in normal growth medium or osteogenic differentiation medium for up to 5 days for cell proliferation ability evaluation, n = 3/group respectively. (E) Young or aged murine BMSC were cultured in presence of DMSO and different concentrations of CB-839 (1, 2 mM) respectively, in osteogenic differentiation medium for 18 days for alizarin red staining and subsequent quantification, n = 5/group respectively. (F) BMM were pretreated with DMSO and different concentrations of CB-839 (1, 2 mM) respectively for 24h, and then subjected to GLS activity assay, n = 6/group respectively. (G) BMM were cultured in the presence of DMSO and different concentrations of CB-839 (1, 2, 4 mM) respectively, in normal growth medium for up to 5 days for cell proliferation ability evaluation, n = 3/group respectively. (H) BMM were cultured in presence of DMSO and different concentrations of CB-839 (1, 2 mM) respectively, with M-CSF and RANKL for 5 days for TRAP staining and subsequent counting of TRAP-positive osteoclasts, n = 5/group respectively. (I) BMM were firstly induced to differentiate into pre-osteoclasts and then digested and seeded on bone slices, treated with DMSO and different concentrations of CB-839 (1, 2 mM) respectively, for osteoclastic resorption activity assay by DAB staining, representative micrographs are shown and scale bar represents 100 mm; total bone resorption area and resorption area per osteoclast were calculated, n = 5/group respectively. Lines and error bars represent mean ± S.D.; *P* values in S5B, S5D, S5G were determined by Kruskal-Wallis test with Dunn's multiple comparisons test, *P* values in S5A by two-way ANOVA with Tukey's multiple comparisons test and the rest by one-way ANOVA with Sidak's multiple comparisons test (* $P < 0.05$, ** $P < 0.01$, *** $P < 0.001$, and **** $P < 0.0001$; "ns" not significant).

© 2024. Guo Q et al. Published online at http://www.aginganddisease.org/EN/10.14336/AD.2024.0201

SUPPLEMENTARY DATA

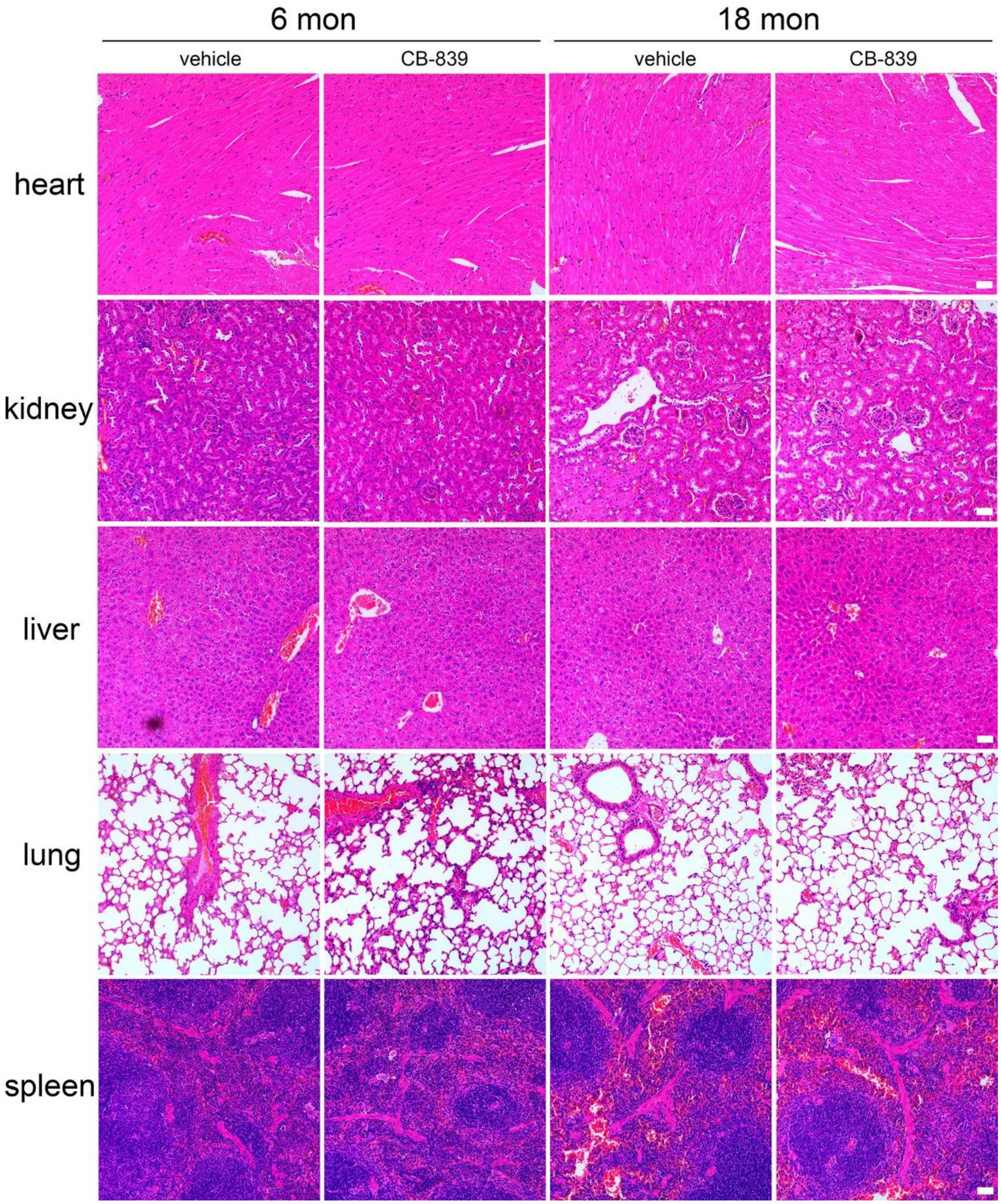

Supplementary Figure 6. Inhibiting GLS via CB-839 doesn't show any significant in vivo toxicity in the mouse model of age-related bone loss. Five organs (lung, heart, kidney, liver, spleen) from each mouse enrolled in 6-month-old and 18-month-old groups treated with CB-839 (200 mg/kg body weight) or same volume of vehicle twice daily were sectioned and hematoxylin-eosin (HE)-stained. Representative images were shown at 200× magnification, scale bars represent 100 mm respectively.

© 2024. Guo Q et al. Published online at http://www.aginganddisease.org/EN/10.14336/AD.2024.0201

SUPPLEMENTARY DATA

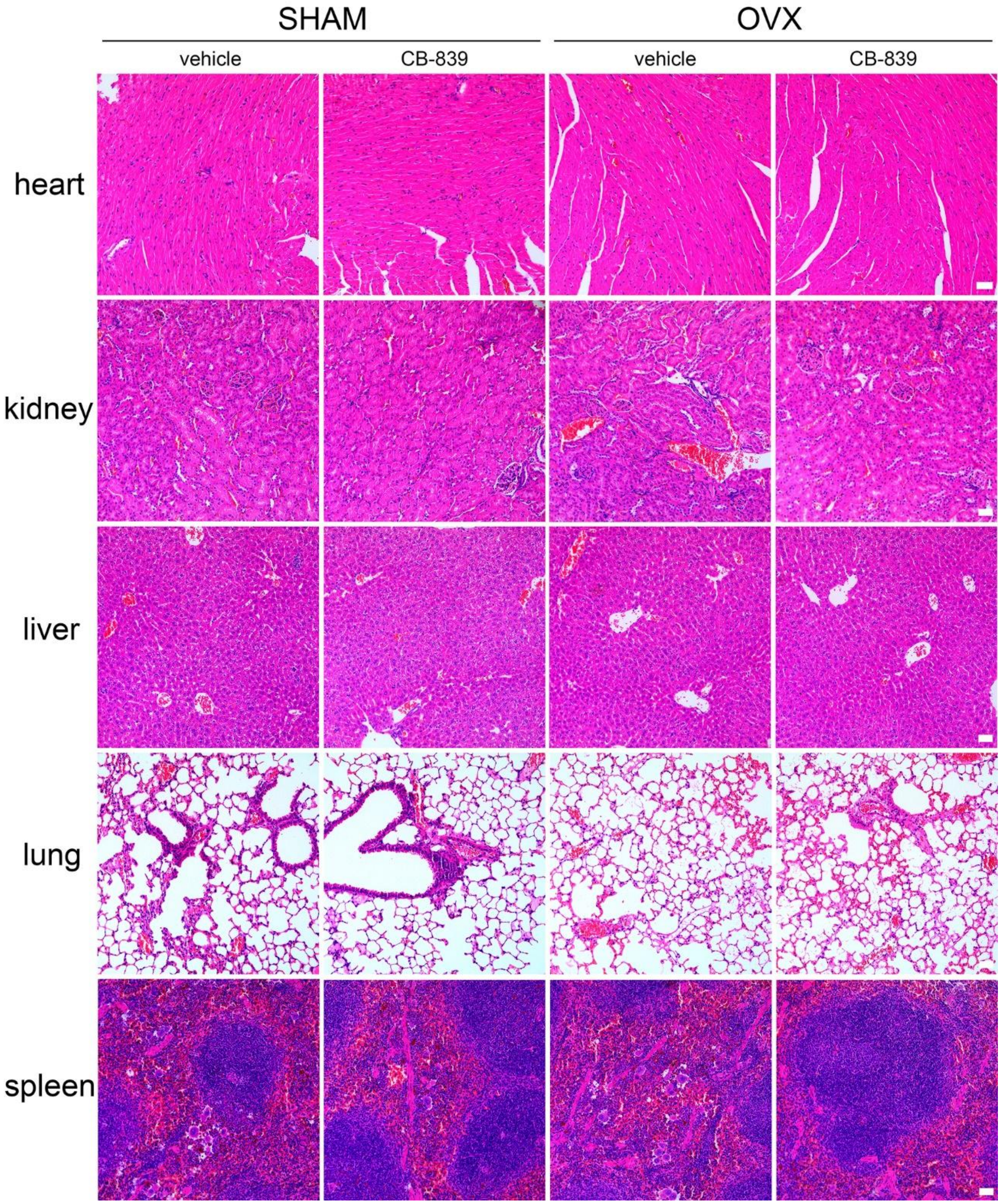

Supplementary Figure 7. Inhibiting GLS via CB-839 doesn't show any significant in vivo toxicity in the mouse model of OVX-induced bone loss. Five organs (lung, heart, kidney, liver, spleen) from sham-operated and ovariectomized groups treated with CB-839 (200 mg/kg body weight) or same volume of vehicle twice daily were sectioned and hematoxylin-eosin (HE)-stained. Representative images were shown at 200× magnification, scale bars represent 100 mm respectively.

© 2024. Guo Q et al. Published online at http://www.aginganddisease.org/EN/10.14336/AD.2024.0201
